# New Target Genes of MITF-Induced microRNA-211 Contribute to Melanoma Cell Invasion

**DOI:** 10.1371/journal.pone.0073473

**Published:** 2013-09-05

**Authors:** Christiane Margue, Demetra Philippidou, Susanne E. Reinsbach, Martina Schmitt, Iris Behrmann, Stephanie Kreis

**Affiliations:** Signal Transduction Laboratory, Life Sciences Research Unit, University of Luxembourg, Luxembourg, Luxembourg; University of Connecticut Health Center, United States of America

## Abstract

The non-coding microRNAs (miRNA) have tissue- and disease-specific expression patterns. They down-regulate target mRNAs, which likely impacts on most fundamental cellular processes. Differential expression patterns of miRNAs are currently being exploited for identification of biomarkers for early disease diagnosis, prediction of progression for melanoma and other cancers and as promising drug targets, since they can easily be inhibited or replaced in a given cellular context. Before successfully manipulating miRNAs in clinical settings, their precise expression levels, endogenous functions and thus their target genes have to be determined. MiR-211, a melanocyte lineage-specific small non-coding miRNA, is located in an intron of TRPM1, a target gene of the microphtalmia-associated transcription factor (MITF). By transcriptionally up-regulating TRPM1, MITF, which is critical for both melanocyte differentiation and survival and for melanoma progression, indirectly drives the expression of miR-211. Expression of this miRNA is often reduced in melanoma samples. Here, we investigated functional roles of miR-211 by identifying and studying new target genes. We show that MITF-correlated miR-211 expression levels are mostly but not always reduced in a panel of 11 melanoma cell lines and in primary and metastatic melanoma compared to normal melanocytes and nevi, respectively. MiR-211 itself only marginally impacted on cell invasion and migration, while perturbation of some new miR-211 target genes, such as AP1S2, SOX11, IGFBP5, and SERINC3 significantly increased invasion. These results and the variable expression levels of miR-211 raise serious doubts on the value of miR-211 as a melanoma tumor-suppressing miRNA and/or as a biomarker for melanoma.

## Introduction

Most likely owing to changed sun-tanning behavior, cutaneous melanoma is rapidly increasing in the industrialized world: an estimated 76,250 people will be diagnosed with invasive melanoma in 2012 in the US, where the incidence increased by 45% from 1992 to 2004 (www.skincancer.org). To this day, molecular markers that would permit differential diagnosis at early stages, classification and prediction of disease progression that could help clinicians to offer more personalized and effective treatment options are not yet consolidated. In this context, miRNAs have been extensively studied to become the sought-after biomarkers, clinical targets, or predictors of cancer and other diseases [1]–[3]. Profiling of miRNA levels and investigation of functional consequences of individual or groups of miRNAs is complicated by their relatively small variations in expression levels (between 1.5–5 fold) [4]. Nevertheless, even small differences in miRNA expression levels might have profound functional consequences for cellular growth, proliferation, differentiation, and other fundamental cellular programs [5]. Another striking denominator of miRNA profiling in many cancers, including melanoma, is the heterogeneity of their basal miRNA levels. The limitations of such profiling studies have recently been reviewed [6] and can be attributed to factors such as technical issues, intrinsic cellular heterogeneity of the clinical samples, different sample types, and the original driving event for development of melanoma (genetic, UV radiation, etc.). Therefore, it remains difficult to this day to summarize existing data in one reliable melanoma-specific miRNA expression set.

Nonetheless, one of few miRNAs that seem specific to the melanocyte lineage is miR-211 [7], [8]. Most studies concur on a down-regulation or a complete loss of this miRNA in the majority of analyzed melanoma patient samples and cell lines when compared to normal skin, nevi or NHEM (normal human melanocytes) [9]–[21]. On the other hand, Mueller et al. did not detect a differential expression of miR-211 in melanoma cell lines versus normal melanocytes [22] and even elevated miR-211 levels in melanoma samples relative to nevi have been described [23]; high expression levels of miR-211 have further been used to discriminate between melanoma and other cancer cell lines [7], [8].

MITF (microphthalmia-associated transcription factor), the master regulator of melanocyte proliferation, survival and differentiation, has intricate regulatory roles in melanoma development [24]. Described as a “lineage addiction” oncogene, MITF is amplified in 10–20% of melanomas; however, MITF has also been attributed tumor-suppressive roles [25]. One of the transcriptional targets of MITF, Melastatin/TRPM1, intronically hosts the gene for miR-211 [11]; hence miR-211 is a direct transcriptional “co-product” of MITF activity.

Here, we report that miR-211 expression was reduced in most but not all melanoma cell lines and patient samples compared to normal melanocytes, confirming the generally reported reduction of miR-211 levels in melanoma samples. We furthermore screened 16 “new” potential target genes, and show that RAB22A, AP1S2, SERINC3 and others are targeted by miR-211. Silencing of the new targets AP1S2, IGFBP5, and SOX11 resulted in reproducible effects on melanoma cell invasion and migration but strikingly, miR-211 alone did not influence proliferative and invasive growth characteristics significantly. Levels of miR-211 always correlated with those of MITF and TRPM1. Therefore, we speculate that MITF is the key factor influencing melanoma cell behavior by directly driving transcription of its melanocyte-lineage specific target genes (MLANA, TYR, TRPM1 etc.) as well as by indirectly up-regulating the intronic miR-211, which in turn down-regulates a number of target genes that may altogether participate in modulating melanoma growth behavior.

## Materials and Methods

### Ethics Statement

The sample collection was approved by the ethical review board of Ethics commission Freiburg (reference EK196/09) and written informed consent was obtained from healthy controls and patients.

### Cell Lines and Cell Culture

Eleven melanoma cell lines were used [26]: Wm35 and Wm9 (paired cell lines, derived from primary and metastatic sites respectively) (Dr. M. Böhm, Münster, Germany), FM55/P and FM55/M1 (paired cell lines, derived from primary and metastatic sites respectively) (European Searchable Tumor Line Database and Cell Bank), IGR39 and IGR37 (paired cell lines, derived from primary and metastatic sites respectively) (Deutsche Sammlung von Mikroorganismen und Zellkulturen), A375 (derived from a metastatic site) (American Type Culture Collection), 1102 (derived from a metastatic site) (Dr. M. Kortylewski, City of Hope, California), MeWo (derived from a metastatic site) (Dr. D. Schadendorf, Essen, Germany) and MelIm (derived from a metastatic site) and MelJuso (derived from a primary tumor) (Dr. A. Bosserhoff, Regensburg, Germany). All cells were maintained in RPMI 1640 supplemented with 10% FCS (PAA Laboratories, Austria), 50 µg/mL penicillin, 100 µg/mL streptomycin, and 0.5 nmol/L L-glutamine. Normal human epidermal melanocytes NHEM-M2 from lightly pigmented adult skin and NHEM-M2-F1, NHEM-M2-F2, NHEM-M2-F3 (PromoCell, Germany), from lightly, medium and darkly pigmented neonatal foreskin, respectively, were maintained in serum- and PMA–free melanocyte growth medium M2 (PromoCell, Germany). Hek293T (Invitrogen) and prostate cancer PC3 cells were grown in DMEM supplemented with 10% FCS, 50 µg/mL penicillin, 100 µg/mL streptomycin and 2.5% Hepes. All cells were grown in a humidified atmosphere with 5% CO_2_ supply and were routinely tested by PCR to be *Mycoplasma* negative. Unless stated otherwise, media and reagents were purchased from LONZA (Belgium).

### Patient Samples

Skin tissue samples from patients with either benign nevi or melanoma were collected at the Dermatology of the University Hospital of Freiburg (Germany) and histopathologically examined to confirm clinical diagnoses. Upon excision, tissues were fixed in FFPE according to standard dermato-histopathologic techniques. In total, 4 pools of benign nevi (RNAs of two different donors each), 9 primary and 12 subcutaneous melanoma metastasis patient samples were analyzed (age and gender information is as described before [26]). Additionally, 2 breast cancer FFPE samples from two patients were included in this study.

### Total RNA Extraction and Quality Control

Total RNA of cell lines was extracted using TRIsure (Bioline USA, Inc.) and treated with DNaseI (New England Biolabs) following each manufacturer’s instructions. For whole-transcriptome microarray analyses, total RNA was extracted using the miRNeasy kit (Qiagen) according to the manufacturer’s protocol with additional on-column DNaseI digestion. For total RNA extraction of FFPE samples, five scalpel-scraped slices of FFPE tissue were pooled and processed using the RT2-FFPE RNA Extraction kit (Qiagen) according to the supplied protocol. Quantity and purity of RNA samples were assessed using a NanoDrop ND-2000 Spectrophotometer.

### Microarrays and Data Analysis

IGR39 cells, seeded at a density of 6×10^4^ cells/well in 12-well culture plates, were transfected with 5 nM of miR-211 mimic or negative control (NCM) as described in the main text. Samples for total RNA extraction were collected 48 h after transfection. RNA quality was assessed using RNA 6000 NanoChips with the Agilent 2100 Bioanalyzer (Agilent, Paolo Alto, USA). Gene expression profiling experiments using GeneChip® Human Gene 1.0 ST arrays and miRNA profiling using GeneChip® miRNA arrays (Affymetrix, Santa Clara, CA, USA) were performed on two independent samples at the CRP-Santé Microarray Unit (Luxembourg) as described before [27] and according to standard protocols. Microarray data are available in the ArrayExpress database (www.ebi.ac.uk/arrayexpress) under accession number E-MEXP-3749. Standard pipeline from the Partek® Genomics SuiteTM (Partek GS) software was used for analysis of data files. Lists of genes were generated by pair-wise comparison of expression data sets (negative control vs. mimic treated samples at 48 h).

### miRNA and mRNA Detection and Relative Quantification by qPCR

Briefly, 250 ng of total RNA from cell lines and FFPE tissues were reverse transcribed in a 10 µl reaction volume with the miScript Reverse Transcription kit (Qiagen) following the supplied protocol. Real-time PCR detection of mRNAs and mature miRNAs was carried out on a CFX96 Detection System (Bio-Rad). For quantification of mature miRNAs, 5 ng RNA input, 2× iQ SYBR Green Supermix (Bio-Rad) and 10× miRNA-specific primer assay (Qiagen) were used. For mRNA detection, 50 ng RNA input, 2× iQ SYBR Green Supermix and 5 pmol gene-specific primer pairs were used (primer sequences are listed in Table S1). Specificity of the qPCR primers was assessed by a post-qPCR melting curve analysis. If not stated otherwise, Ct-values for mRNA and miRNA species were normalized to three reference genes: TBP (TATA binding protein), HPRT1 (Hypoxanthine phosphoribosyltransferase 1) and cyclophilin A for mRNAs and RNU1A, RNU5A (RNA, U1A/5A small nuclear) and SCARNA17 (small Cajal body-specific RNA 17) for mature miRNAs. Based on the geometric mean of the three reference genes, a normalization factor was calculated for each sample using geNorm, a VBA applet for Microsoft Excel. The relative amount of each target in each sample was then corrected by dividing its amount by the corresponding normalization factor. Fold changes were calculated by dividing the normalized relative amount of treated samples with the normalized relative amount of the control sample. Statistical significance was assessed with t-test or one-way ANOVA followed by a Dunnett Post-Hoc test. P values of <0.05 (*), <0.01 (**) and <0.001 (***) were considered significant.

### Western Blot Analysis

Western blots were performed as described before [27] to detect successful transfection and subsequent expression of the MITF protein in A375 cells and following siRNA-mediated silencing of MITF in FM55/M1 cells. The following primary antibodies were used: anti-MITF (Clone C5, Sigma-Aldrich, 1∶500), anti-RAB22A (Proteintech, 1∶2000) and anti-α-tubulin (Santa Cruz, 1∶2500). HRP-labeled secondary antibodies were purchased from Cell Signaling Technology (Boston, MA).

### MITF Transient Transfection

A375 cells were seeded at a density of 3×10^5^ cells/well in 6-well culture plates (Greiner) and were transfected 24 h later with 1 µg of pCMV-SPORT6-MITF expressing vector (cDNA clone MGC:75121 IMAGE:6066096; Open Biosystems) using the TransIT-LT1 (Mirus) transfection reagent. RNA and protein lysates were harvested at 24 and 48 h after transfection and processed as described in Supplementary Materials and Methods.

### Oligonucleotide Transfections

5×10^4^ cells/well were seeded in 6-well culture plates (Greiner) and transfected after 24 h with 5 nM miRNA (10 nM for NHEMs) mimic or mimic negative control (NCM) or 75 nM ON-TARGET siRNA or siRNA negative control (siCtrl) using the HiPerfect transfection reagent according to the manufacturer’s protocol (Qiagen), with the exception of MITF siRNA for which 10^5^ cells/well were seeded in 6-well plates and Lipofectamine™ RNAiMAX was used as transfection reagent following the supplied guidelines (Life Technologies). Treated cells were assayed 24, 48 and 72 h after transfection. MiRNA mimics were purchased from Qiagen and all ON-TARGET siRNAs from Dharmacon.

### Dual Luciferase Reporter Gene Assays

Partial sequences of the RAB22A and AP1S2 3′UTRs containing putative miR-211 target sites were PCR-amplified from A375 cDNA (ThermoScript RT kit, Invitrogen) and cloned into the pmirGLO Dual Luciferase miRNA target expression vector (Promega) downstream of the firefly luciferase gene. The miR-211 full complementary (FC) (positive control) sequence as well as the RAB22A, AP1S2, SERINC3, KCNMA1, M6PR, SSRP1, PDE3A, LIFR, IGFBP5, SOX11 and SOX4 predicted miR-211 binding sites were cloned individually into the pmirGLO vector (primer and oligonucleotide sequences are listed in Table 1). A375 cells were seeded at a density of 5×10^4^ cells/well in 24-well plates 24 h prior to transfection. Cells were transiently co-transfected with 500 ng plasmid and 5 nM miR-211 mimic or negative control for 48 and 72 h using Dharmafect Duo (Thermo Scientific). Samples were lysed with 1× Passive Lysis Buffer (Promega) and luciferase activities were measured consecutively using the Dual Luciferase Reporter Assay System (Promega) according to the manufacturer’s instructions. The Firefly/Renilla activity ratios of mimic-treated samples were calculated and normalized to the respective ratios of the negative control-treated samples for each construct and each time point. Significance was assessed by a paired t-test.

**Table 1:** Summary of miR-211 target genes.

Targets	miR-211 binding sites ^#^	mRNA levels aftermiR-211 M treatment##	Luciferase assays	Effect of target genesilencing on MIG and INV			mRNA levels afterMITF silencing	Target function	Ref.
**own data in melanoma cells:**									
**RAB22A**	3 c +2 pc bs	↓↓↓	√√√	–	–		↑	GTPase, endosomal trafficking	[34] 1
**AP1S2**	2 c bs	↓↓↓	√√	↑↑↑	↑↑↑		–	member of adaptin proteins,endosomal trafficking	
**SERINC3**	1 pc bs	↓↓↓	√	–	↑↑		↑↑	serine incorporator, transmembrane protein	
**M6PR**	1 c +1 pc bs	↓↓	√√	/	/		↓	membrane receptor, endosomaltrafficking	
**IGFBP5**	2 c +2 pc bs	↓	√√	↑↑↑	↑↑↑		/	IGF-binding protein	
**PDE3A**	1 pc bs	↓	√√	–	↑		/	phosphodiesterase	
**SSRP1**	1 c bs	↓↓	√√√	–	↑		–	subunit of FACT (chromatin transcr. elongation factor)	
**SOX11**	3 c bs	–	√√	↑	↑↑↑		/	TF	
**LIFR**	1 pc bs	–	√√√	/	/		/	cytokine receptor	
**SOX4**	1 c +1 pc bs	/	√√	/	/		/	TF	
**NR3C1**	3 c +1 pc bs	↓↓	/	/	/		/	TF, glucocorticoid receptor	
**ANGPT1**	1 c +2 pc bs	↓↓ (+/−)	/	/	/		/	GF	[35] 2
**SERP1**	2 pc bs	–	/	/	/		/	ER protein	
**SNAI2**	1 pc bs	–	/	/	/		/	transcriptional repressor	[34] 1
**ATL3**	-	↓ (+/−)	/	/	/		/	ER GTPase	
**ADAMTS1**	-	–	/	/	/		/	metalloproteinase	
**KCNMA1**	2 pc bs	protein and mRNA levelsdown (stable cell lines)*	√	/	↓(stable shRNA )*		/	Ca^2+^-channel	[10] 3
**previously described miR-211 targets:**									
**TGFβR2**	1 c bs	↓*	√*	/		↓*	/	GF receptor	[11]
**NFAT5**	1 c bs	↓*	√*	/		↓*	/	TF	[11]
**IGF2R**	1 c bs	↓*	–*	/		↓*	/	GF receptor	[11]
**BRN-2**	2 pc bs	only protein levels down, (stable cell lines)*	√*	/		/	/	TF	[12]
**PRAME**	1 pc bs	protein levels down*	√*	/		/	/	transcriptional repressor	[17]
**CHD5**	1 c +1 pc bs	protein levels down,(stable cell lines)*	√*	/		/	/	helicase DNA-binding protein	[30]
**RUNX2**	1 c +3 pc bs	protein levels down,(stable cell lines)*	√*	/		/	/	TF	[36]
**CREB5**	2 c +2 pc bs	↓*	/	/		/	↑*	TF	[34]
**ELOVL6**	1 c +2 pc bs	↓*	/	/		/	↑*	fatty acid elongase	[34]
**TCF-12**	1 c +2 pc bs	↓*	/	/		/	↑*	TF	[34]
**IL11**	2 pc bs	↓*	√*	/		/	/	cytokine	[37]
**MMP9**	1 pc bs	protein and mRNA levels down (after overexpr.)*	/	/		/	/	matrix metalloproteinase	[38]
**DDIT3**	-	↓*	√*	/		/	/	transcriptional regulator	[39]

Abbreviations: M, mimic; INV, invasion; MIG, migration; Ref., reference; c, conserved; pc, poorly conserved; bs, binding site; TF, transcription factor; GF, growth factor; ER, endoplasmic reticulum; # according to TargetScan;

## determined by qPCR; +/−, depending on cell line;/, not analysed; –, no effect; ↑, increased; ↓, decreased; √, decrease in luciferase activity (1/2/3 symbols, minor/intermediate/major effects);

* data from literature (not quantified);

1 in fetal retinal pigment epithelium cells;

2 in lung carcinoma cells;

3 in melanoma cells.

### Invasion and Migration Assays

Different melanoma cell lines, seeded at a density of 2×10^4^ cells/well in collagen I-coated (200 µg/ml, Millipore) 96-well plates (Essen Imagelock, Essen Bioscience), were transfected after 24 h with 25 nM miR-211 mimic or NCM, or with 375 nM siRNA or the negative siCtrl as described above. 24 h after transfection, when cells had reached 80–90% confluence, a wound was scratched across each well with the Cellplayer 96-well woundmaker (Essen Bioscience). To study invasion, cells were covered with 50 µl type I collagen solution (1 mg/ml collagen in normal growth medium) that was allowed to gel for 30 min at 37°C. This matrix was overlaid with additional 100 µl normal growth medium. To study migration, 150 µl normal growth medium (without collagen) was added to the cells. Wound conﬂuency was monitored with the Incucyte LiveCell Imaging System (Essen Bioscience) by measuring cell confluency every 3 h for a total of 72 h; it was quantified with the relative wound density v1.0 metrics of the included analysis software: invasion was monitored in wells containing a collagen matrix while wells without collagen were used for analysis of migration events. 24, 48 and 72 h after scratching, RNA was extracted from transfected cells (from 4 pooled 96-wells) to determine expression levels of miR-211 or the silenced target genes by qPCR. Wells with inappropriate or uneven scratches were excluded from analysis.

## Results

In order to identify miRNAs differentially expressed in melanoma cells, we have performed miRNA microarray analyses using IGR39, IGR37 (primary and metastatic melanoma cell lines derived from the same patient) and NHEM (normal human melanocytes). We have previously reported on several of those differentially expressed miRNAs [26]. The growing number of studies suggesting an important role of miR-211 in melanoma cells led us to re-analyze our miRNA array data. Interestingly, miR-211 levels were undetectable in NHEM and IGR39, while a strong expression was scored in IGR37 metastatic cells. These unexpected results contradicted some previously published data and prompted us to investigate miR-211 expression levels and target genes in more detail.

### Expression Profiling of miR-211

**Figure 1:**
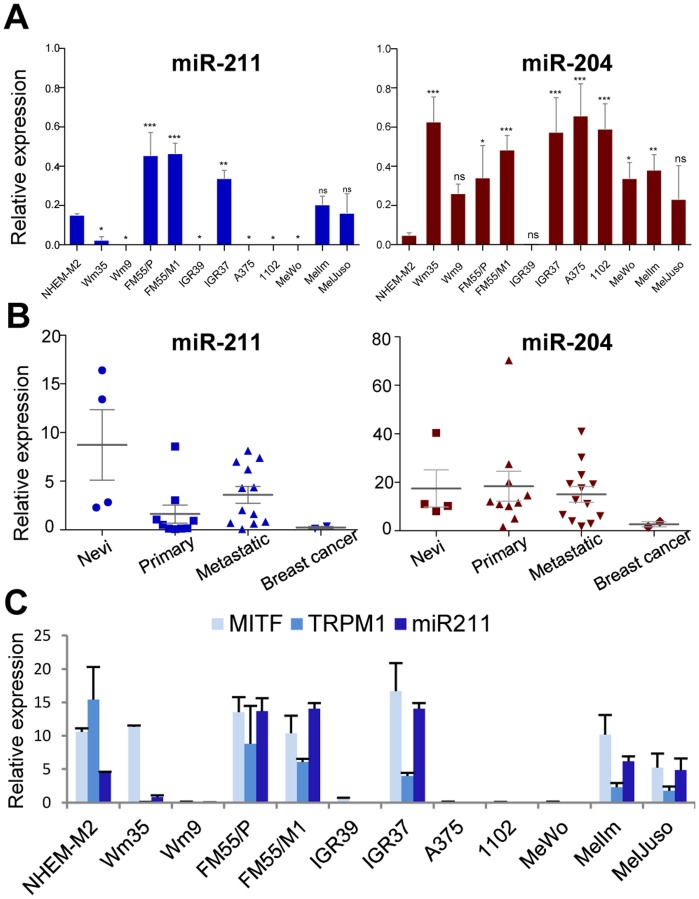
Expression profiling of miR-211 and co-expressed proteins. (A) RNA of primary melanocytes (NHEM-M2) and 11 different melanoma cell lines was analyzed for relative miR-211 (blue) and miR-204 (red) expression levels by qPCR. Statistical significance was assessed with ANOVA (repeated measures) followed by a Dunnett Post-Hoc multiple comparison test. P values of <0.05 (*), <0.01 (**) and <0.001 (***) were considered significant. (B) RNA of FFPE patient samples from 4 nevi, 9 primary and 12 metastatic melanoma samples and 2 breast cancer samples were analyzed as above. (C) Co-expression of MITF, TRPM1 and the intronic miR-211 was confirmed in the same cell lines and statistical significance was tested as in (A). Except for FFPE patient samples, all experiments were performed at least in biological triplicates.

MiR-211 is located in intron 6 of the TRPM1 gene on chromosome 15. Interestingly, miR-204, which shares the same seed region with miR-211 and therefore many predicted target genes, lies in intron 6 of TRPM3, which also encodes a calcium channel protein with similar functions to TRPM1. Because of this intriguing connection, we initially profiled both miRNAs. Figure 1 illustrates completely different expression profiles for both miRNAs in melanoma cell lines and patient samples. Six of 11 tested cell lines had absent or very low levels of miR-211 compared to NHEM; however, IGR37 (which had been used in our initial miRNA arrays) and FM55/P and FM55/M1 (primary and metastatic tumors from the same patient) had much higher levels of miR-211 than NHEM, indicating that there is intrinsic variation in the expression levels of this miRNA (Figure 1A). Additionally, we detected a general down-regulation of miR-211, but not of miR-204, in samples from primary and metastatic melanoma patients as compared to nevi. Both miR-211 and miR-204 were not expressed in 2 breast cancer samples (Figure 1B).

The correlation and inter-dependency of MITF, TRPM1 and miR-211 has been demonstrated before [10]–[12]. We confirm this strong correlation in 10 of 11 tested melanoma cell lines, which clearly supports the idea that MITF drives expression of TRPM1 and by doing so it up-regulates miR-211, an intronic passenger of this gene (Figure 1C). MITF over-expression in cells with very low endogenous MITF induced expression of TRPM1, miR-211 and of MLANA, a direct target gene of MITF, which was used to control for functionality of transfected MITF (Figure S1A). Vice versa, silencing of MITF in cells, which normally express this transcription factor, clearly reduced expression of TRPM1, miR-211, and MLANA (Figure S1B). These results again confirm that miR-211 expression is strongly dependent on functional MITF. Therefore, it is the absence or presence of functional MITF in a given cell line or patient sample that determines the amounts of miR-211 and consequently expression levels of miR-211 target genes.

### Identification of miR-211 Target Genes

**Figure 2:**
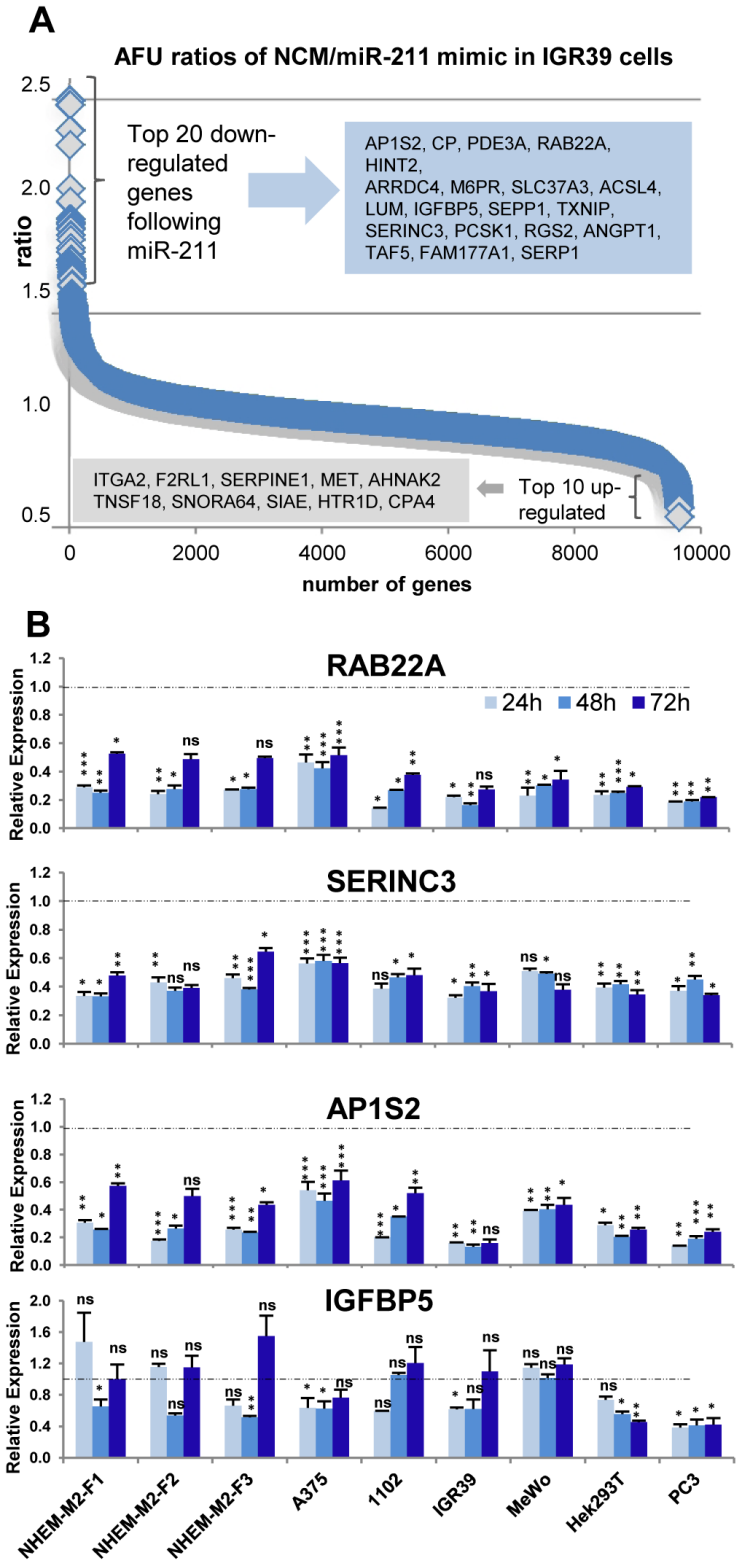
Identification of miR-211 target genes. (A) To identify new miR-211 target genes, miR-211-negative IGR39 melanoma cells were transfected with 5 nM miR-211 mimic or negative control (NCM) for 48 h. RNAs from two independent samples were extracted and analyzed separately by Affymetrix mRNA arrays. The scatter plots show ratios of normalized and averaged arbitrary fluorescence units (AFU) from two independent arrays between NCM- and mimic-treated cells for all mRNAs. Top-regulated genes are listed in grey boxes. (B) Expression levels (qPCR) of 4 selected and putative miR-211 targets RAB22A, SERINC3, AP1S2 and IGFBP5 after 5 nM miR-211 mimic or NCM (or 10 nM for primary melanocytes NHEM) treatment for 24, 48 and 72 h +/− SEM are shown. Levels of NCM-treated cells were set to 1. Significance was tested with a t-test and p values are as described in Figure 1.

To obtain an overview of those potential target genes, whose mRNA is degraded or down-regulated by miR-211 (opposed to targets for which only protein levels would be altered), we transfected IGR39 melanoma cells with miR-211 mimics or negative controls and analyzed samples by mRNA arrays. Figure 2A shows that expression levels of the vast majority of mRNAs was not or only marginally altered following miR-211 mimic treatment, while some were strongly down- or up-regulated indicating a potential connection with miR-211 levels. Focusing on down-regulation events, we compiled a list of tentative targets from our own array analysis, from literature and using several miRNA target gene prediction algorithms (Table 1).

Next, we analyzed by qPCR expression changes of selected putative target genes following miR-211 mimic treatment in 6 different cell lines and NHEM samples from 3 different donors (Figure 2B) after thorough optimization of miR-211 mimic concentrations: titration experiments over a period of 72 h showed that concentrations of 5 nM miR-211 mimic were sufficient to induce significant effects on target gene expression, exemplified by robust down-regulations of RAB22A, SERINC3 and AP1S2 over time (Figure S2). Consequently, 5 nM miR-211 mimics were applied throughout the study to all cancer cell lines (10 nM was used for primary NHEM cells). Figure 2B depicts strong down-regulatory effects of miR-211 mimic on mRNA levels of the newly identified putative RAB22A, SERINC3 and AP1S2 target genes in different cell lines and NHEMs, which became visible after 24 h and remained stable for up to 3 days. Effects on IGFBP5 (insulin-like growth factor binding protein 5) were generally less consistent in melanoma cells but significant in prostate cancer cells (PC3). Eleven additional potential targets have been analyzed by qPCR after mimic treatment (data not shown) and results are summarized in Table 1.

To further analyze corresponding protein levels, we tested several commercially available antibodies against the new miR-211 targets however, except for RAB22A (Figure S2), antibodies were mostly unspecific and could not be used for reliable detection and quantification of protein levels (data not shown).

Next, we reduced endogenous MITF levels by siRNA treatment in order to verify the correlation of MITF and miR-211 levels followed by analysis of mRNA levels for selected target genes (Figure S3). However, only RAB22A and SERINC3 levels increased simultaneously to a decrease in miR-211 due to silencing of MITF suggesting a direct interaction between miR-211 and these target genes. Even though we only used melanoma cell lines, the different experimental approaches demonstrate that results vary with respect to individual target genes. The interpretation of whether a mRNA is directly targeted by a given miRNA does not only depend on the cellular background but even more so on experimental assays and conditions.

### Direct Interaction of miR-211 with Target Gene mRNAs

**Figure 3:**
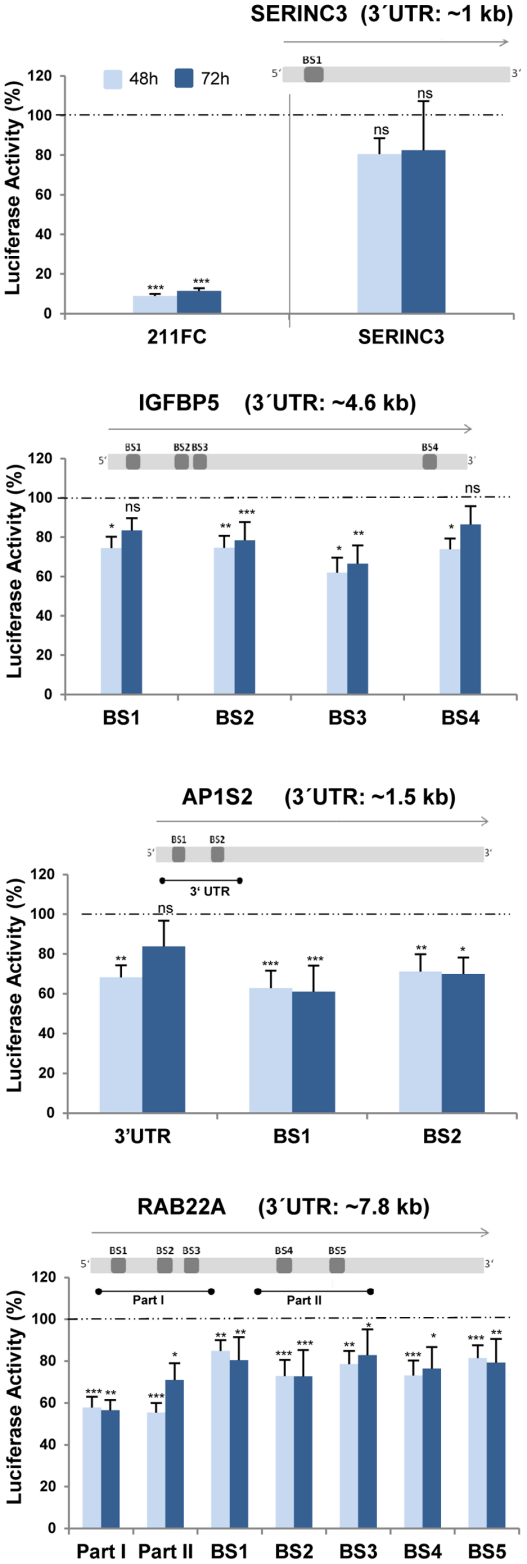
Luciferase reporter gene assays confirm new direct targets of miR-211 in melanoma cells. A luciferase reporter vector containing indicated stretches of the target 3′UTR or single binding sites (BS) for miR-211 (depicted by schemes above the graphs) were transfected together with 5 nM miR-211 mimic or NCM into A375 melanoma cells. After 48 and 72 h, luciferase activity was measured. The full complementary (FC) sequence of miR-211 was cloned into the luciferase vector and served as a positive control. Shown are ratios of mimic/NCM-treated cells. Activity of NCM-treated cells was set to 1 with bars representing the average of at least 3 biological replicates per time point, +/− SEM. Significance was tested by a paired t-test with p values as described above.

To determine whether the above-observed effects are due to direct interactions between miRNA-211 and its target mRNAs in melanoma cells, we performed extensive luciferase reporter gene assays in A375 cells with partial 3′UTRs and individually-cloned single miR-211 binding sites of the respective targets. Regarding the targets SERINC3, IGFBP5, the GTPase RAB22A and the adaptin AP1S2, luciferase activity decreased after transfection of miR-211 mimics compared to negative controls (NCM) (Figure 3). Additional target genes for which we have observed decreased luciferase activity are shown in Figure S4. KCNMA1 has previously been described as a direct miR-211 target in A375 cells [10]. However, in our hands and applying a dual luciferase reporter gene assay, only non-significant effects of miR-211 on this calcium channel gene were scored (Figure S4).

### Effects of miR-211 and its Target Genes on Melanoma Cell Invasion and Migration

**Figure 4:**
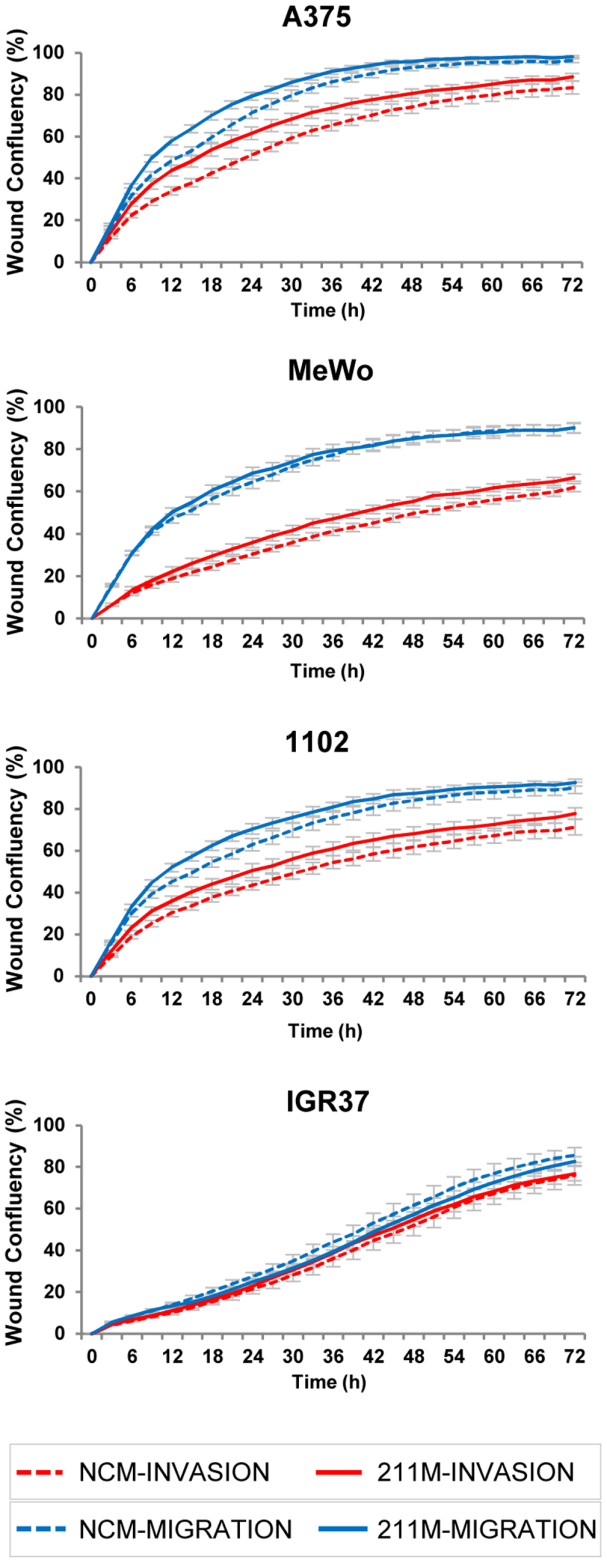
MiR-211 has no significant effects on melanoma cell invasion and migration. Four melanoma cell lines were transfected with miR-211 mimic (solid lines) or NCM (dotted lines); successful mimic transfection was confirmed by qPCR in all samples (Figure S5). After 24 h, a scratch/wound assay was performed and cells were grown for up to 72 h. To study invasion (red lines), cells were covered with a collagen matrix after wound scratching; to study migration (blue lines), cells were grown in wells without a collagen matrix containing only normal growth medium. Invasion and migration were monitored by measuring wound closure every 3 h for a total of 72 h with the Incucyte LiveCell Imaging System (Essen Bioscience). Basal levels of miR-211 in these cell lines are shown in Figure 1. The graphs depict averages from two biological replicates each including at least quadruplicate samples +/−SEM. In cells without endogenous miR-211 (MeWo, 1102, and A375), the mimic treatment caused a very small but reproducible increase in invasion; in IGR37 (high endogenous miR-211), a further increase of miR-211 by transfection did not affect invasion/migration at all.

Interestingly, several groups have previously described negative effects of miR-211 on invasion and/or migration of melanoma cells [10]–[12]. In order to reproduce and further investigate these suppressive effects on invasion, we introduced miR-211 mimics into several melanoma cell lines with high or absent levels of endogenous miR-211 (Figure 1A). Surprisingly, miR-211 mimic treatment did not reduce cell invasion and/or migration in all tested cell lines, but rather increased invasion slightly but reproducibly (Figure 4 and S5). Migration of melanoma cell lines was also only marginally altered by miR-211 mimics; cell proliferation was not affected at all (data not shown).

**Figure 5:**
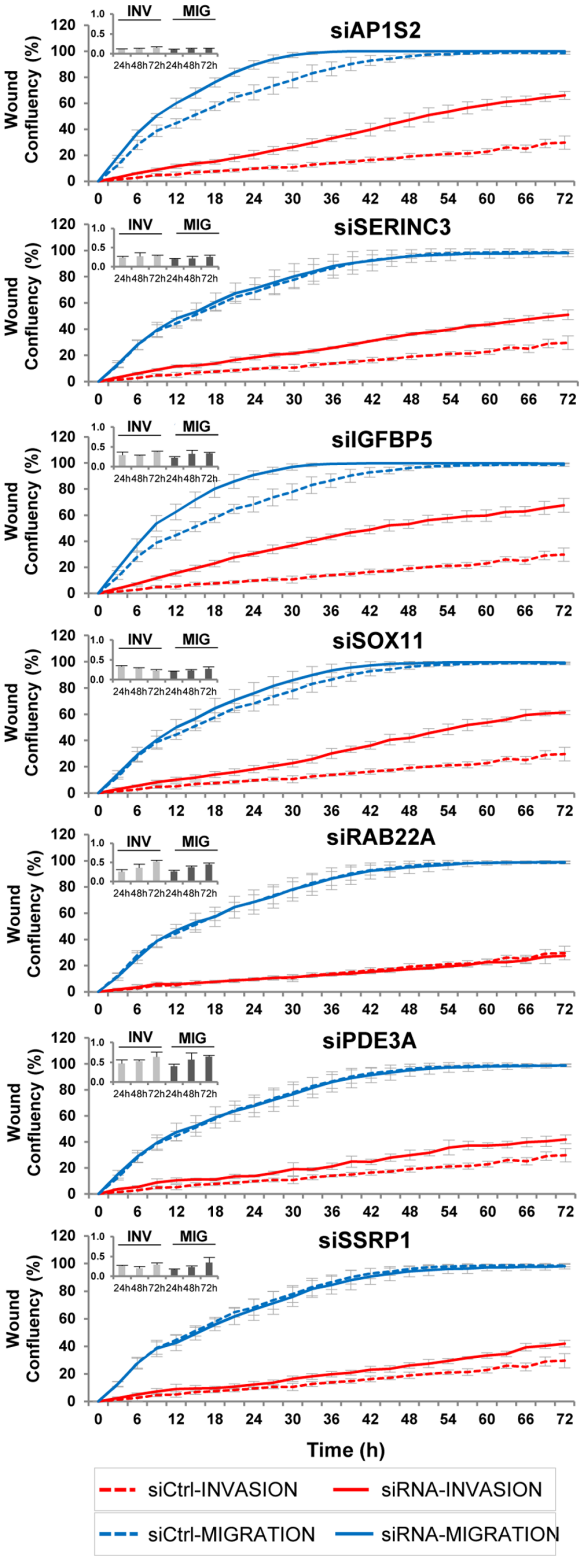
siRNA-mediated down-regulation of new miR-211 targets has an impact on melanoma cell invasion and migration. A375 cells were transfected with siRNAs directed against selected miR-211 targets. After 24 h, a scratch/wound assay was performed as described in Figure 4. To ensure efficient siRNA-mediated down-regulation of target mRNAs, qPCR was performed on total RNA (extracted from 4 pooled wells for each treatment) at 24, 48 and 72 h after transfection in the invasion and migration wells (small inlets, upper left corners). Representative graphs of four biological replicate experiments are shown. Error bars show STD from at least 4 technical replicates for each measurement.

Finally, we investigated a potential involvement of the newly identified, individual miR-211 target genes in migration and invasion of melanoma cells. Intriguingly, siRNA-mediated ablation of AP1S2, IGFBP5, SERINC3 and SOX11 significantly increased invasion by up to 40%. Migration was augmented up to 30 h of assay time for only AP1S2 and IGFBP5 in A375 melanoma cells (Figure 5). Inhibition of RAB22A, the most prominent direct miR-211 target (Table 1) did, however, not influence cell growth characteristics. Silencing of PDE3A and SSRP1 had only minor effects on cell invasion and none on migration. Table 1 summarizes our own data on 16 putative miR-211 target genes that we have identified and compiled using microarray data from miR-211 mimic-treated melanoma cell lines, experimental data from different assay formats and several target gene prediction software tools (TargetScan, Pictar, MiRanda, DIANA). To complete this comprehensive miR-211 target gene summary, we also listed 13 previously described targets from different cell lines and tissues. Taken together, we identified and characterized several genes that are specifically down-regulated by miR-211, a miRNA, which is transcriptionally induced by MITF. We only observed minor or no effects of miR-211 itself on growth behavior of melanoma cells, challenging previous notions of a strong suppressive effect of miR-211 on cell invasion. Nevertheless, inhibition of some of the new target genes had robust and reproducible effects on the invasive behavior of melanoma cells.

## Discussion

MicroRNAs are major players in post-transcriptional regulation of tissue- and disease-specific gene expression. The importance of miRNAs as master gene regulators, as potential diagnostic markers for many different diseases and as targets for future therapeutic applications form the basis for ongoing research on the relevance of miRNAs in melanoma and other cancers [3].

A number of recent studies have reported modified expression levels of miR-211 in melanoma cells and/or have investigated the functional roles of miR-211 and its target genes [10]–[12], [17], [18]. A clear correlation has been demonstrated between MITF and miR-211, which we also confirm here: MITF transcriptionally activates, amongst many other genes, the calcium channel TRPM1 (melastatin) and by doing so, the intronic miR-211 is simultaneously up-regulated. In the majority of melanoma cell lines and patient samples, MITF and miR-211 levels seem reduced compared to normal melanocytes or nevi. However and irrespective of their invasive capacities, a considerable proportion of melanoma samples (in our study nearly 50%, Figure 1) have high MITF and correspondingly high miR-211 expression levels compared to healthy cells [10], [16]. We show that miR-211 expression was indeed reduced but only in 6 of 11 melanoma cell lines and in most but not all melanoma patient samples. For all tested cell lines (except Wm35), we found that miR-211 levels correlated with MITF and thus TRPM1 levels (Figure 1C, Figure S1). In this context, the initially described tumor-suppressive and anti-invasive properties of TRPM1 [28] have recently been shown to be executed by its intronic miR-211 (and not by TRPM1 itself) via down-regulation of the miR-211 direct and indirect targets TGFBR2, NFAT5, and IGF2R [11].

To date, only few target genes of miR-211 in melanoma cells have been described. For instance, the miR-211-mediated down-regulation of the calcium ion-regulated potassium channel KCNMA1, whose expression is up-regulated in metastatic melanoma, was reported to account for the anti-invasive effects of this miRNA [10]. Here, using a dual luciferase assay, KCNMA1 was also regulated by miR-211 in the same cell line (A375), albeit to a much lower extent. The transcription factor BRN-2, which can repress MITF expression, is also targeted by miR-211. It was reasoned that this negative feedback loop on MITF, enforced by the loss of miR-211, would keep melanoma cells in a de-differentiated, pro-invasive state [12].

Although previous studies have reported tumor-suppressing traits of miR-211 by decreasing invasion [11], [12], we show here that miR-211 itself hardly had any measurable and reproducible effects on invasion or migration (Figure 4), for which we used a next generation live Imaging system (IncuCyte). The IncuCyte monitors long-term cellular growth, behavior, and morphology in a 96-well format allowing for accurate statistical analysis of total or single invasion or migration events. When introducing miR-211 mimics, growth behavior was not significantly altered in a melanoma cell line that endogenously expresses this miRNA while small pro-invasive and pro-migratory effects were scored in 3 melanoma cell lines that do not express endogenous miR-211. In agreement with this, Boyle and colleagues reported that over-expression of miR-211 reduced invasion only in MITF- and thus miR-211-positive cells, however, when cells expressing low levels of MITF and miR-211 were transfected with miR-211 mimic, the authors observed increased invasion of the melanoma cells [12]. In this context, some reports have also stated oncogenic roles for miR-211 in non-melanocytic tissues: in oral carcinoma, miR-211 expression was correlated with poor prognosis [29] and Cai et al. demonstrated that miR-211 promoted colorectal cancer cell growth in vitro and in vivo [30]. Most cancers become life-threatening once they have metastasized and therefore, the ability to invade surrounding tissue represents one of the key events determining the fate of a cancer patient [31]. Concerning the role of MITF, we have initial data indicating reduced invasion and migration of IGR37 melanoma cells following MITF silencing by siRNAs (Figure S6). Proliferation, however, was neither influenced in MITF-silenced cells nor in miR-211 mimic-treated cells (data not shown). Therefore, it will be crucial to more precisely define the role of MITF and miR-211 in order to consolidate the true relevance of this microRNA for melanoma cell invasion.

Several new miR-211 targets were identified: RAB22A, AP1S2, SERINC3, IGFBP5, PDE3A, M6PR, SSRP1 and SOX11 were generally down-regulated by miR-211, however, the degree of regulation depended on the experimental assays. Upon silencing of several of these target genes, we observed clearly enhanced invasive properties, suggesting that high levels of miR-211 would indeed favor an invasive phenotype. However, miR-211 itself did not modulate growth characteristics of several melanoma cell lines. This suggests that either miR-211 alone has no ability to actively perturb cell invasion, migration and proliferation or other yet unidentified miR-211 targets counteract the effect of the mimic in our experimental setup or the action of several miRNAs with similar functional targets would be required to achieve a robust effect. In this context, we subjected the new and previously described miR-211 target genes to Ingenuity pathway analysis (IPA) but no common functional pathways were found indicating independent roles or a yet unknown interplay of these candidates in melanoma cells. Nevertheless, 3 of the top 4 miR-211 targets appear to be involved in endosomal trafficking (Table 1), a common function worth pursuing.

As has been comprehensively reviewed by several authors [6], [19], [25], [32], MITF not only is the master regulator of healthy melanocytes, it also plays important roles in melanoma development. Depending on MITF expression levels, melanoma cells can switch between proliferative and invasive programs [33]. However, many different known and unknown microenvironmental as well as genetic factors upstream or downstream of MITF can influence its activity and target gene selectivity. If we can determine more precisely what influences different MITF levels in melanoma or if we could modulate MITF levels themselves, we might be able to better predict the behavior and progression of melanoma. The complex interplay of MITF with its direct targets, together with MITF-dependent up-regulation of miR-211, which in turn targets several genes that partake in important cellular processes, is likely to determine melanoma cell fate. Since effects of isolated miR-211 itself and some of its individual target genes are rather small, it is reasonable to assume that only the combination of effects is sufficient to alter growth and proliferation characteristics of melanoma cells.

## Supporting Information

Figure S1 **MiR-211 expression is driven by MITF.** (A) A375 melanoma cells, which have no endogenous MITF (Figure 1), were transfected with a MITF expression vector (+MITF) or with a negative control (ctrl). After 24 and 48 h, RNA was extracted and expression levels of MITF, MLANA and TRPM1 (direct MITF targets) and miR-211 were analyzed by qPCR. MLANA, TRPM1 and miR-211 were undetectable in ctrl-treated cells. Western Blots (below) confirmed over-expression of MITF. (B) Silencing of MITF was performed in FM55/M1 melanoma cells (with high levels of endogenous MITF, Figure 1) with siRNA directed against MITF (+siMITF) or a negative control siRNA (+NC). After 24, 48 and 72 h RNA was extracted and analyzed as in A). Western blots confirmed efficient down-regulation of MITF protein expression after 24 h up to 72 h. For qPCR, an average of 4 biological replicates each consisting of 2 technical replicates are shown (except for TRPM1 detection after MITF transfection, for which 2 biological replicates were analyzed). A paired t-test was used to determine significance and p values of <0.05 (*), <0.01 (**) and <0.001 (***) were considered significant. These results confirm that MITF drives TRPM1 and subsequently the expression of intronic miR-211 as has been shown before [11].
(PPTX)

Figure S2 **Titration of miR-211 mimic and NCM concentrations followed by analysis of putative miR-211 target expression levels.** A375 melanoma cells were transfected with different concentrations of miR-211 mimics and NCM for 24, 48 and 72 h; concentrations ranged from 50 nM to 0.5 nM. A Western blot showing biological duplicates of miR-211-mediated down-regulation of RAB22A is depicted below the bar chart (RAB22A was the only potential target, for which a specific antibody was available). The top graph shows tracking of miR-211 after over-expression and below, different graphs depict mRNA expression levels of selected putative miR-211 targets (RAB22A, AP1S2, SERINC3). Levels of NCM-treated cells were set to 1. Average of 3 biological replicates +/− SEM are shown. Statistical analysis of miR-211 tracking: repeated measures ANOVA followed by Bonferroni Post-Hoc Multiple Comparison test (NCM vs 211 M); targets: paired t-test (NCM vs 211 M per time point per amount). P values of <0.05 (*), <0.01 (**) and <0.001 (***) were considered significant.
(PPTX)

Figure S3 **siRNA-mediated ablation of MITF shows effects on two miR-211 target genes.** FM55/M1 melanoma cells with high endogenous MITF levels were treated for 24, 48 and 72 h with siRNA directed against MITF. RNA was extracted and miR-211 as well as selected target gene mRNA levels were analyzed by qPCR. Blue bars depict target gene mRNA levels, while the black line shows endogenous mir-211 expression levels measured in the same samples. Average of 4 biological replicates each consisting of 2 technical replicates is shown +/− SEM of negative ctrl vs. siMITF); significance was tested with a paired t-test (p values as above). Only RAB22A and SERINC3 levels show a negative correlation with lowered miR-211 amounts due to silencing of MITF, suggesting a direct interaction between miR-211 and these two target genes. Although AP1S2, M6PR and SSRP1 were directly targeted by miR-211 in reporter gene assays, in this experimental setup their expression levels showed no obvious inverse correlation with miR-211 levels.
(PPTX)

Figure S4 **Luciferase reporter gene assays confirm more new direct targets of miR-211 in melanoma cells.** A luciferase reporter vector containing the single binding sites (BS) for miR-211 of the target genes was transfected together with 5 nM mimic or NCM into A375 cells. After 48 and 72 h, luciferase activity was measured. The different graphs show luciferase activity in targets with 1, 2 or 3 binding sites for miR-211. Depicted are the ratios of mimic/NCM treated cells. Activity of NCM treated cells was set to 1, and averages of at least 3 biological replicates per time point, +/− SEM are shown. Significance was tested with a paired t-test with p values as described before. SSRP1, PDE3A, LIFR, M6PR, SOX4 and SOX11 have single, two or three 3′UTR binding sites for miR-211 and all seem to be direct targets of miR-211 with more or less significant down-regulations following 211-mimic treatment. KCNMA1, a previously described direct target of miR-211 [10], has two binding sites, both of which seem to be targeted by miR-211, albeit with low efficiency.
(PPTX)

Figure S5 **Tracking of mimic transfections.** Four melanoma cell lines were transfected with miR-211 mimic (red lines) or NCM (blue lines) and migration and invasion was measured as shown in Figure 4. Successful mimic transfection was confirmed by qPCR in all samples with grey bars representing NCM control transfections and blue bars showing miR-211 mimic transfections in either invasion (light blue) or migration (dark blue) experiments.
(PPTX)

Figure S6 **siRNA-mediated ablation of MITF influences melanoma cell invasion and migration.** A) To ensure efficient siRNA-mediated down-regulation of MITF and its targets, qPCR was performed on total RNA (extracted from 4 pooled wells for each treatment) at 24, 48 and 72 h after transfection in the invasion and migration wells. B) IGR37 melanoma cells with high endogenous MITF levels were transfected with siRNAs directed against MITF. After 24 h, a scratch/wound assay was performed as described in Figure 4. Representative graphs of three biological replicate experiments are shown. Error bars show STD from at least 4 technical replicates for each measurement.
(PPTX)

Table S1 **Oligonucleotides and Primers for 3′ UTR cloning (Luciferase assays) and qPCR Primers.**
(DOCX)
