# Supplementary material for: New Target Genes of MITF-Induced microRNA-211 Contribute to Melanoma Cell Invasion
Source: PLoS One. 2013 Sep 5;8(9):e73473. doi: 10.1371/journal.pone.0073473 (PMC3764006; doi:10.1371/journal.pone.0073473)
Supplement: Figure S1 — MiR-211 expression is driven by MITF. (A) A375 melanoma cells, which have no endogenous MITF (Figure 1), were transfected with a MITF expression vector (+MITF) or with a negative control (ctrl). After 24 and 48 h, RNA was extracted and expression levels of MITF, MLANA and TRPM1 (direct MITF targets) and miR-211 were analyzed by qPCR. MLANA, TRPM1 and miR-211 were undetectable in ctrl-treated cells. Western Blots (below) confirmed over-expression of MITF. (B) Silencing of MITF was performed in FM55/M1 melanoma cells (with high levels of endogenous MITF, Figure 1) with siRNA directed against MITF (+siMITF) or a negative control siRNA (+NC). After 24, 48 and 72 h RNA was extracted and analyzed as in A). Western blots confirmed efficient down-regulation of MITF protein expression after 24 h up to 72 h. For qPCR, an average of 4 biological replicates each consisting of 2 technical replicates are shown (except for TRPM1 detection after MITF transfection, for which 2 biological replicates were analyzed). A paired t-test was used to determine significance and p values of <0.05 (*), <0.01 (**) and <0.001 (***) were considered significant. These results confirm that MITF drives TRPM1 and subsequently the expression of intronic miR-211 as has been shown before [11]. (PPTX) [file pone.0073473.s001.pptx]

## Slide 1
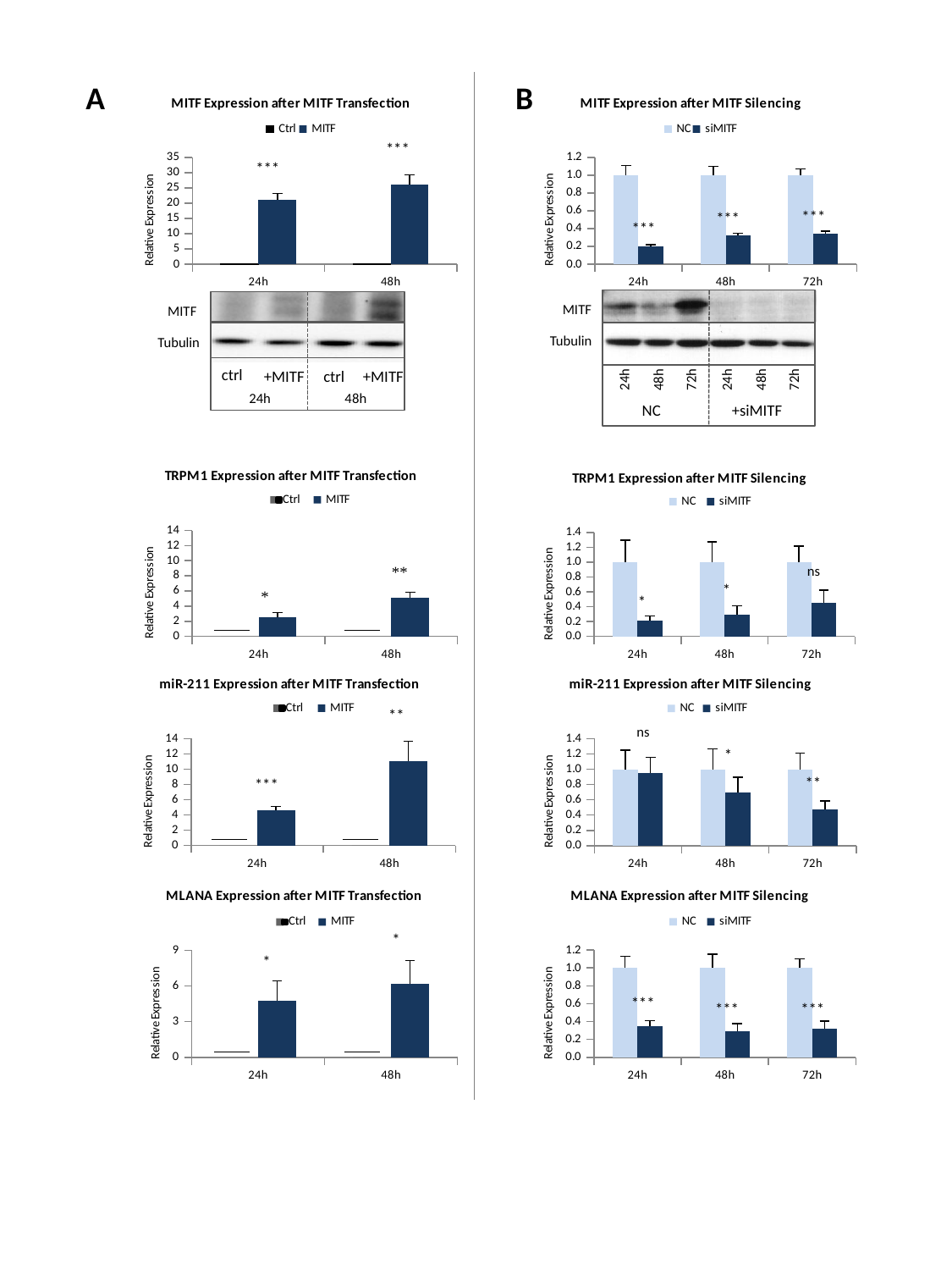

A
B
### Chart: MITF Expression after MITF Transfection
| Category | | |
|---|---|---|
| 24h | 0.13067910749459183 | 21.20128892906803 |
| 48h | 0.08768655873497275 | 26.20487029858473 |***
***
### Chart: MITF Expression after MITF Silencing
| Category | | |
|---|---|---|
| 24h | 0.9999999999999999 | 0.20317774962915938 |
| 48h | 1.0 | 0.3246930745890045 |
| 72h | 0.9999999999999999 | 0.34419933221356774 |***
***
***
MITF
MITF
Tubulin
Tubulin
24h
48h
72h
24h
48h
72h
ctrl
+MITF
ctrl
+MITF
24h
48h
NC
+siMITF
### Chart: TRPM1 Expression after MITF Transfection
| Category | | |
|---|---|---|
| | 0.0 | 2.5078952109053647 |
| | 0.0 | 5.084438982921859 |
### Chart: TRPM1 Expression after MITF Silencing
| Category | | |
|---|---|---|
| 24h | 1.0 | 0.21148671992365445 |
| 48h | 1.0 | 0.2909109233136792 |
| 72h | 1.0 | 0.4522048964862906 |ns
*
*
### Chart: miR-211 Expression after MITF Transfection
| Category | | |
|---|---|---|
| | 0.0 | 4.608954632047686 |
| | 0.0 | 11.074696198063245 |**
***
### Chart: miR-211 Expression after MITF Silencing
| Category | | |
|---|---|---|
| 24h | 1.0 | 0.9459755645340073 |
| 48h | 0.9999999999999999 | 0.6961182300567462 |
| 72h | 0.9999999999999999 | 0.46916136988474394 |ns
*
**
### Chart: MLANA Expression after MITF Silencing
| Category | | |
|---|---|---|
| 24h | 1.0 | 0.34504858317313797 |
| 48h | 0.9999999999999999 | 0.2909279873598001 |
| 72h | 0.9999999999999999 | 0.3187916985627168 |***
***
***
### Chart: MLANA Expression after MITF Transfection
| Category | | |
|---|---|---|
| | 0.0 | 4.7508739272076985 |
| | 0.0 | 6.152600533281466 |*
*
