# Supplementary material for: New Target Genes of MITF-Induced microRNA-211 Contribute to Melanoma Cell Invasion
Source: PLoS One. 2013 Sep 5;8(9):e73473. doi: 10.1371/journal.pone.0073473 (PMC3764006; doi:10.1371/journal.pone.0073473)
Supplement: Figure S2 — Titration of miR-211 mimic and NCM concentrations followed by analysis of putative miR-211 target expression levels. A375 melanoma cells were transfected with different concentrations of miR-211 mimics and NCM for 24, 48 and 72 h; concentrations ranged from 50 nM to 0.5 nM. A Western blot showing biological duplicates of miR-211-mediated down-regulation of RAB22A is depicted below the bar chart (RAB22A was the only potential target, for which a specific antibody was available). The top graph shows tracking of miR-211 after over-expression and below, different graphs depict mRNA expression levels of selected putative miR-211 targets (RAB22A, AP1S2, SERINC3). Levels of NCM-treated cells were set to 1. Average of 3 biological replicates +/− SEM are shown. Statistical analysis of miR-211 tracking: repeated measures ANOVA followed by Bonferroni Post-Hoc Multiple Comparison test (NCM vs 211 M); targets: paired t-test (NCM vs 211 M per time point per amount). P values of <0.05 (*), <0.01 (**) and <0.001 (***) were considered significant. (PPTX) [file pone.0073473.s002.pptx]

## Slide 1
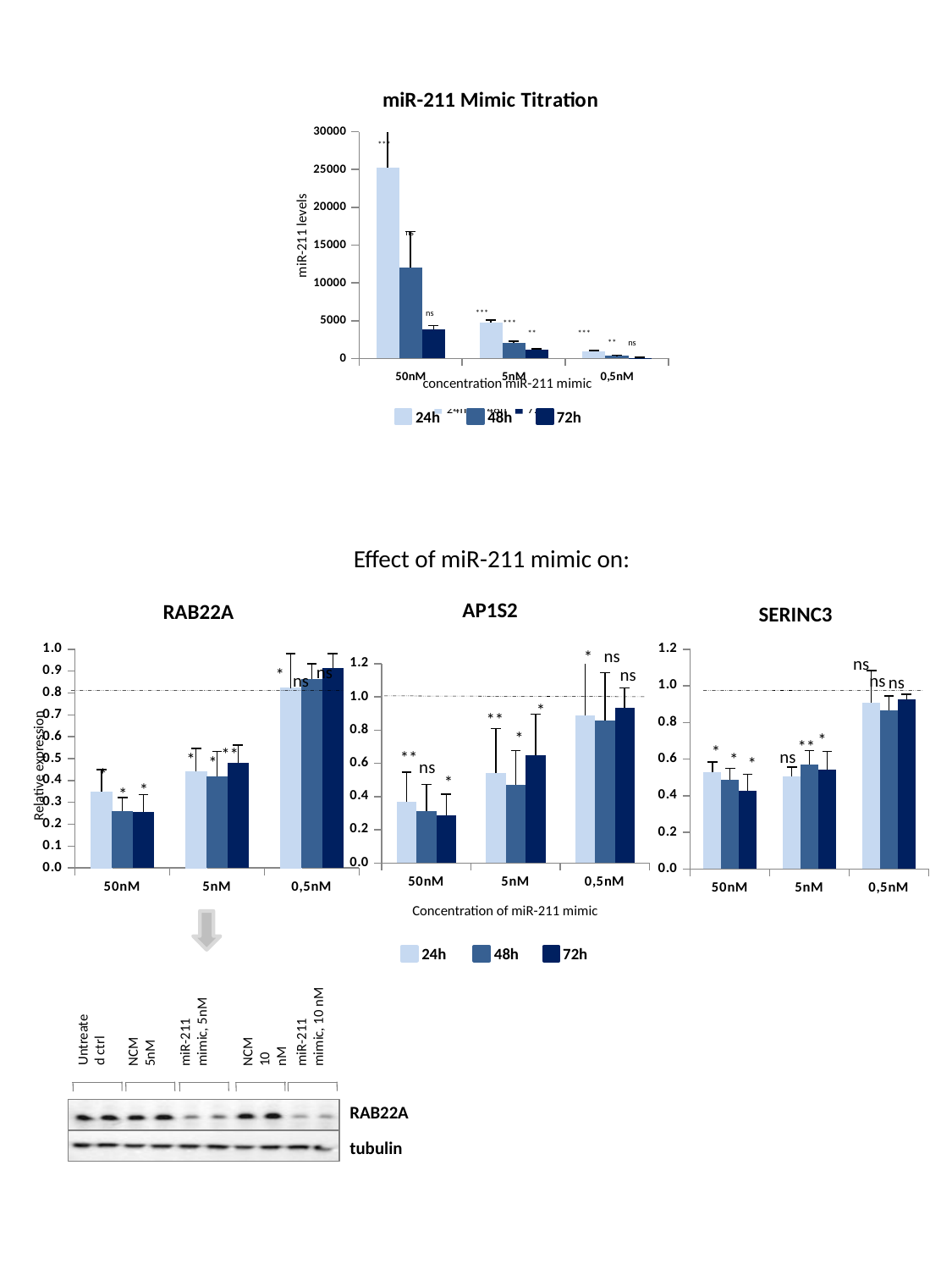

### Chart: miR-211 Mimic Titration
| Category | 24h | 48h | 72h |
|---|---|---|---|
| 50nM | 25239.679995965882 | 11993.167701621807 | 3839.2371691540848 |
| 5nM | 4726.988607187246 | 2004.856107850168 | 1139.63852134962 |
| 0,5nM | 938.955342078543 | 338.9511344867835 | 94.86993140253034 |***
miR-211 levels
ns
ns
***
***
**
***
**
ns
concentration miR-211 mimic
24h
48h
72h
Effect of miR-211 mimic on:
AP1S2
RAB22A
SERINC3
### Chart
| Category | 24h | 48h | 72h |
|---|---|---|---|
| 50nM | 0.3662403070040373 | 0.31248786812147994 | 0.2881360315471157 |
| 5nM | 0.5397162900464837 | 0.4689898277612378 | 0.6485226433184575 |
| 0,5nM | 0.8904706901785217 | 0.8599754036162048 | 0.9338815748945286 |
### Chart
| Category | 24h | 48h | 72h |
|---|---|---|---|
| 50nM | 0.3485229931600122 | 0.26179665660367024 | 0.25790186232024914 |
| 5nM | 0.44075949226541666 | 0.41731605328406296 | 0.48273431752629026 |
| 0,5nM | 0.8217029758019412 | 0.8642384235173896 | 0.9153794184913326 |
### Chart
| Category | 24h | 48h | 72h |
|---|---|---|---|
| 50nM | 0.5266222528512717 | 0.4870303583910176 | 0.4288578889724477 |
| 5nM | 0.5038346852235317 | 0.5709485029756497 | 0.543918213167998 |
| 0,5nM | 0.9096707188282337 | 0.8642758493276577 | 0.9250172459779479 |ns
*
ns
ns
*
ns
ns
ns
ns
*
**
*
*
**
*
**
ns
**
*
*
*
*
ns
Relative expression
*
*
*
*
Concentration of miR-211 mimic
24h
48h
72h
miR-211 mimic, 5nM
miR-211 mimic, 10 nM
Untreated ctrl
NCM 5nM
NCM 10 nM
RAB22A
tubulin
