# Supplementary material for: New Target Genes of MITF-Induced microRNA-211 Contribute to Melanoma Cell Invasion
Source: PLoS One. 2013 Sep 5;8(9):e73473. doi: 10.1371/journal.pone.0073473 (PMC3764006; doi:10.1371/journal.pone.0073473)
Supplement: Figure S3 — siRNA-mediated ablation of MITF shows effects on two miR-211 target genes. FM55/M1 melanoma cells with high endogenous MITF levels were treated for 24, 48 and 72 h with siRNA directed against MITF. RNA was extracted and miR-211 as well as selected target gene mRNA levels were analyzed by qPCR. Blue bars depict target gene mRNA levels, while the black line shows endogenous mir-211 expression levels measured in the same samples. Average of 4 biological replicates each consisting of 2 technical replicates is shown +/− SEM of negative ctrl vs. siMITF); significance was tested with a paired t-test (p values as above). Only RAB22A and SERINC3 levels show a negative correlation with lowered miR-211 amounts due to silencing of MITF, suggesting a direct interaction between miR-211 and these two target genes. Although AP1S2, M6PR and SSRP1 were directly targeted by miR-211 in reporter gene assays, in this experimental setup their expression levels showed no obvious inverse correlation with miR-211 levels. (PPTX) [file pone.0073473.s003.pptx]

## Slide 1
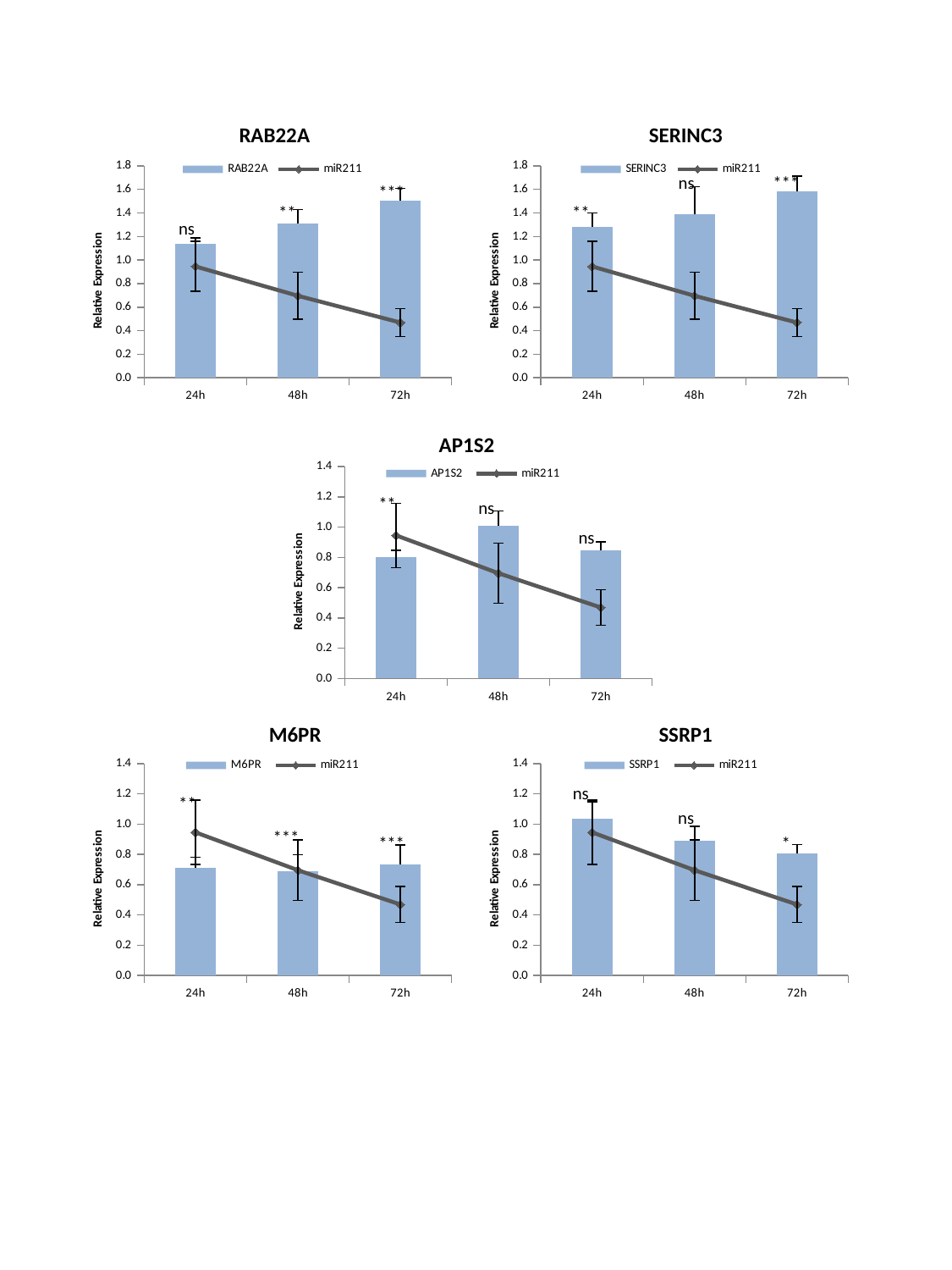

RAB22A
SERINC3
### Chart
| Category | | |
|---|---|---|
| 24h | 1.1352744001319337 | 0.9459755645340072 |
| 48h | 1.3115914995734694 | 0.6961182300567462 |
| 72h | 1.5056221795919862 | 0.4691613698847439 |***
**
ns
### Chart
| Category | | |
|---|---|---|
| 24h | 1.2832798024631538 | 0.9459755645340072 |
| 48h | 1.3861532340295324 | 0.6961182300567462 |
| 72h | 1.581172988148338 | 0.4691613698847439 |ns
***
**
AP1S2
### Chart
| Category | | |
|---|---|---|
| 24h | 0.8017751440664836 | 0.9459755645340072 |
| 48h | 1.009415008578764 | 0.6961182300567462 |
| 72h | 0.8443210284848557 | 0.4691613698847439 |**
ns
ns
M6PR
SSRP1
### Chart
| Category | | |
|---|---|---|
| 24h | 0.7088045531680108 | 0.9459755645340072 |
| 48h | 0.68992395289984 | 0.6961182300567473 |
| 72h | 0.734994618512975 | 0.4691613698847439 |**
***
***
### Chart
| Category | | |
|---|---|---|
| 24h | 1.037914304303304 | 0.9459755645340072 |
| 48h | 0.8891900315710812 | 0.6961182300567462 |
| 72h | 0.8045647302702126 | 0.4691613698847439 |ns
ns
*
