# Supplementary material for: New Target Genes of MITF-Induced microRNA-211 Contribute to Melanoma Cell Invasion
Source: PLoS One. 2013 Sep 5;8(9):e73473. doi: 10.1371/journal.pone.0073473 (PMC3764006; doi:10.1371/journal.pone.0073473)
Supplement: Figure S4 — Luciferase reporter gene assays confirm more new direct targets of miR-211 in melanoma cells. A luciferase reporter vector containing the single binding sites (BS) for miR-211 of the target genes was transfected together with 5 nM mimic or NCM into A375 cells. After 48 and 72 h, luciferase activity was measured. The different graphs show luciferase activity in targets with 1, 2 or 3 binding sites for miR-211. Depicted are the ratios of mimic/NCM treated cells. Activity of NCM treated cells was set to 1, and averages of at least 3 biological replicates per time point, +/− SEM are shown. Significance was tested with a paired t-test with p values as described before. SSRP1, PDE3A, LIFR, M6PR, SOX4 and SOX11 have single, two or three 3′UTR binding sites for miR-211 and all seem to be direct targets of miR-211 with more or less significant down-regulations following 211-mimic treatment. KCNMA1, a previously described direct target of miR-211 [10], has two binding sites, both of which seem to be targeted by miR-211, albeit with low efficiency. (PPTX) [file pone.0073473.s004.pptx]

## Slide 1
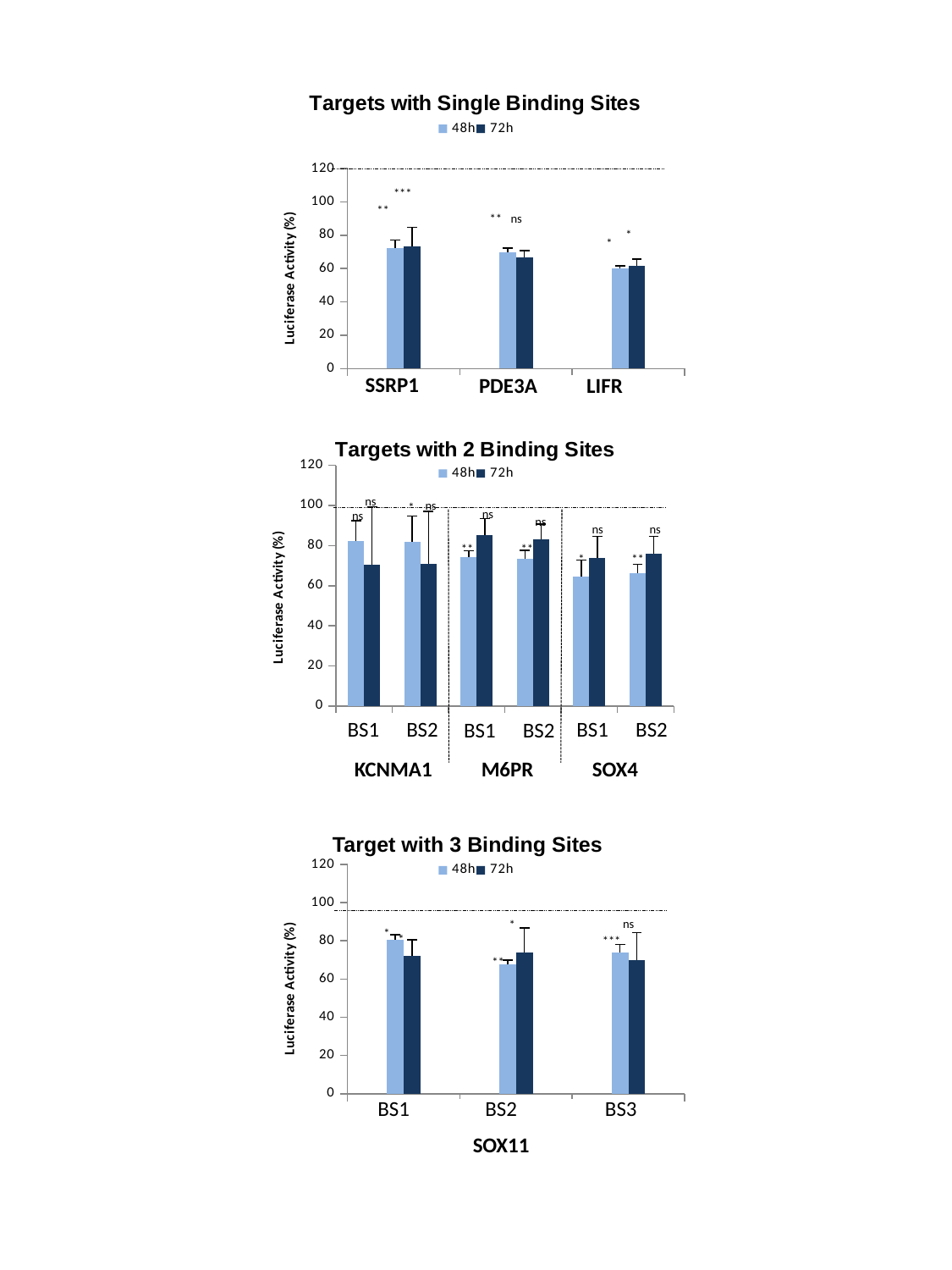

### Chart: Targets with Single Binding Sites
| Category | | |
|---|---|---|
| SSRP1 | 72.09020724353465 | 73.27272695392786 |
| PDE3A | 69.94230309904123 | 66.84168985408988 |
| LIFR | 60.33349653239843 | 61.6716871040831 |***
**
**
ns
*
*
SSRP1
PDE3A
LIFR
### Chart: Targets with 2 Binding Sites
| Category | | |
|---|---|---|
| KCNMA1-BS1 | 82.20375857958064 | 70.34215969847813 |
| KCNMA1-BS2 | 82.03169958569453 | 70.99649339347155 |
| M6PR-BS1 | 74.1986570654131 | 85.34374528305294 |
| M6PR-BS2 | 73.20211337022293 | 83.25306357616068 |
| SOX4-BS1 | 64.71808617245586 | 73.9607935917678 |
| SOX4-BS2 | 66.4209344854129 | 76.1350338776006 |ns
ns
*
ns
ns
ns
ns
ns
**
**
*
**
BS1
BS2
BS1
BS2
BS1
BS2
KCNMA1
M6PR
SOX4
### Chart
| Category | | |
|---|---|---|
| BS1 | 80.72495253441089 | 72.35187758151382 |
| BS2 | 67.85664911833321 | 73.99805231627265 |
| BS3 | 73.91322309517302 | 70.06954977218521 |ns
*
*
*
***
**
Target with 3 Binding Sites
BS1
BS2
BS3
SOX11
