# Supplementary material for: New Target Genes of MITF-Induced microRNA-211 Contribute to Melanoma Cell Invasion
Source: PLoS One. 2013 Sep 5;8(9):e73473. doi: 10.1371/journal.pone.0073473 (PMC3764006; doi:10.1371/journal.pone.0073473)
Supplement: Figure S5 — Tracking of mimic transfections. Four melanoma cell lines were transfected with miR-211 mimic (red lines) or NCM (blue lines) and migration and invasion was measured as shown in Figure 4. Successful mimic transfection was confirmed by qPCR in all samples with grey bars representing NCM control transfections and blue bars showing miR-211 mimic transfections in either invasion (light blue) or migration (dark blue) experiments. (PPTX) [file pone.0073473.s005.pptx]

## Slide 1
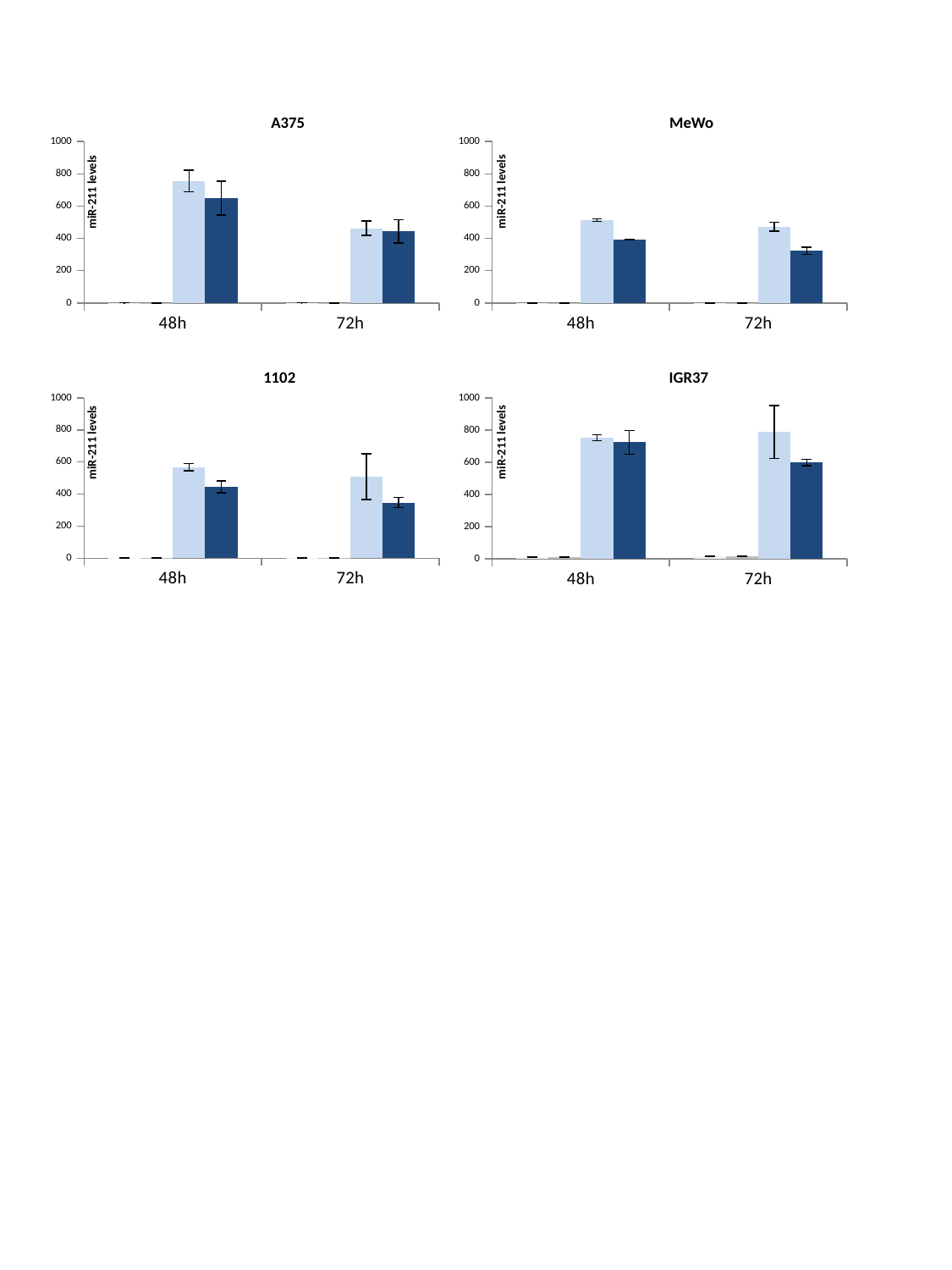

A375
### Chart
| Category | | | | |
|---|---|---|---|---|
| 48h | 1.3036610374772284 | 0.3906989768995907 | 756.2950819595337 | 650.0536440478478 |
| 72h | 0.7041151012529224 | 0.1826941757301334 | 462.5491825097116 | 443.9997234096257 |MeWo
### Chart
| Category | NCM INV | NCM MIG | | |
|---|---|---|---|---|
| 48h | 0.27090317074036147 | 0.1227987255664913 | 513.8053056581284 | 393.44004640973105 |
| 72h | 0.17947498282952373 | 0.07741972157437017 | 471.5435815976747 | 322.17428677015346 |miR-211 levels
miR-211 levels
1102
IGR37
### Chart
| Category | | | | |
|---|---|---|---|---|
| 48h | 0.9977254976807197 | 0.3006756048833798 | 568.3179398074486 | 444.95096555075986 |
| 72h | 1.3767231106209084 | 0.5561485241429378 | 508.09436128195557 | 346.83978105507515 |
### Chart
| Category | NCM INV | NCM MIG | 211M INV | 211M MIG |
|---|---|---|---|---|
| 48h | 10.41298576566309 | 11.231790331480028 | 752.3272817043028 | 723.484152414403 |
| 72h | 14.271627671211867 | 16.54273155841164 | 787.4046454885571 | 598.5900596447625 |miR-211 levels
miR-211 levels
