# Supplementary material for: New Target Genes of MITF-Induced microRNA-211 Contribute to Melanoma Cell Invasion
Source: PLoS One. 2013 Sep 5;8(9):e73473. doi: 10.1371/journal.pone.0073473 (PMC3764006; doi:10.1371/journal.pone.0073473)
Supplement: Figure S6 — siRNA-mediated ablation of MITF influences melanoma cell invasion and migration. A) To ensure efficient siRNA-mediated down-regulation of MITF and its targets, qPCR was performed on total RNA (extracted from 4 pooled wells for each treatment) at 24, 48 and 72 h after transfection in the invasion and migration wells. B) IGR37 melanoma cells with high endogenous MITF levels were transfected with siRNAs directed against MITF. After 24 h, a scratch/wound assay was performed as described in Figure 4. Representative graphs of three biological replicate experiments are shown. Error bars show STD from at least 4 technical replicates for each measurement. (PPTX) [file pone.0073473.s006.pptx]

## Slide 1
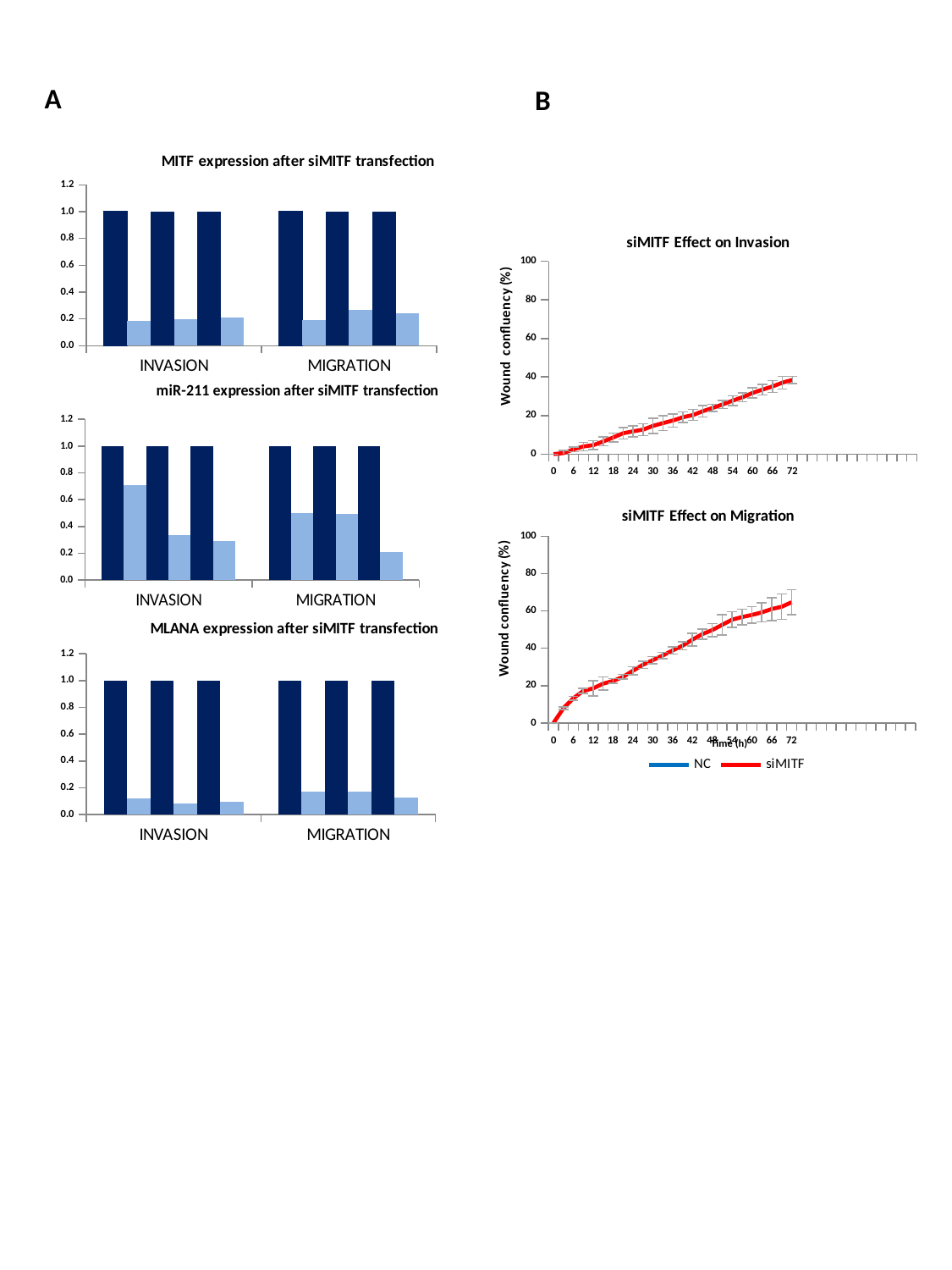

A
B
### Chart: MITF expression after siMITF transfection
| Category | NC-24h | siMITF-24h | NC-48h | siMITF-48h | NC-72h | siMITF-72h |
|---|---|---|---|---|---|---|
| INVASION | 1.0 | 0.18556544629485341 | 1.0 | 0.19682699413904714 | 1.0 | 0.2094967837396713 |
| MIGRATION | 1.0 | 0.19411721863447817 | 1.0 | 0.26425451024862895 | 1.0 | 0.23981602983636757 |
### Chart: miR-211 expression after siMITF transfection
| Category | NC-24h | siMITF-24h | NC-48h | siMITF-48h | NC-72h | siMITF-72h |
|---|---|---|---|---|---|---|
| INVASION | 1.0 | 0.7071067811865475 | 1.0 | 0.33680839421642345 | 1.0 | 0.2911833966171138 |
| MIGRATION | 1.0 | 0.5 | 1.0 | 0.49654624771851874 | 1.0 | 0.20877197985709273 |
### Chart: MLANA expression after siMITF transfection
| Category | NC-24h | siMITF-24h | NC-48h | siMITF-48h | NC-72h | siMITF-72h |
|---|---|---|---|---|---|---|
| INVASION | 1.0 | 0.11907974973381685 | 1.0 | 0.08333116731682247 | 1.0 | 0.09705860940880406 |
| MIGRATION | 1.0 | 0.17253966907166665 | 1.0 | 0.16840419717721872 | 1.0 | 0.13 |
### Chart: siMITF Effect on Invasion
| Category | NC | siMITF |
|---|---|---|
| 0 | 0.0 | 0.0 |
| | 0.644 | 0.5316666666666665 |
| 6 | 2.3579999999999997 | 2.4133333333333336 |
| | 3.71 | 3.9299999999999997 |
| 12 | 4.976000000000002 | 4.670000000000001 |
| | 6.1718 | 6.556666666666668 |
| 18 | 8.122000000000002 | 8.653333333333334 |
| | 9.616000000000001 | 10.823333333333332 |
| 24 | 12.344000000000001 | 11.823333333333332 |
| | 15.102 | 12.700000000000001 |
| 30 | 17.759999999999994 | 14.633333333333333 |
| | 21.02 | 16.066666666666666 |
| 36 | 23.62 | 17.433333333333323 |
| | 27.139999999999997 | 19.13333333333333 |
| 42 | 29.979999999999997 | 20.233333333333324 |
| | 33.86 | 22.233333333333324 |
| 48 | 37.2 | 23.96666666666667 |
| | 40.0 | 25.766666666666666 |
| 54 | 42.74000000000001 | 27.733333333333324 |
| | 45.2 | 29.5 |
| 60 | 47.52 | 31.700000000000003 |
| | 49.4 | 33.43333333333333 |
| 66 | 51.74000000000001 | 35.06666666666664 |
| | 53.72000000000001 | 37.03333333333333 |
| 72 | 56.0 | 38.46666666666665 |
### Chart: siMITF Effect on Migration
| Category | NC | siMITF |
|---|---|---|
| 0 | 0.0 | 0.0 |
| | 5.317999999999999 | 7.886666666666668 |
| 6 | 8.262 | 13.200000000000001 |
| | 12.906000000000002 | 17.13333333333333 |
| 12 | 12.820000000000002 | 18.533333333333324 |
| | 15.66 | 21.033333333333324 |
| 18 | 18.759999999999994 | 22.46666666666667 |
| | 21.660000000000004 | 24.63333333333333 |
| 24 | 24.68 | 27.900000000000002 |
| | 29.759999999999998 | 31.13333333333333 |
| 30 | 34.34 | 33.6 |
| | 39.040000000000006 | 36.06666666666664 |
| 36 | 44.82000000000001 | 38.80000000000001 |
| | 50.22000000000001 | 41.333333333333336 |
| 42 | 55.9 | 44.60000000000001 |
| | 62.14 | 47.60000000000001 |
| 48 | 68.17999999999999 | 49.733333333333334 |
| | 72.58 | 52.53333333333333 |
| 54 | 75.7 | 55.333333333333336 |
| | 78.96000000000002 | 56.73333333333335 |
| 60 | 82.36 | 57.833333333333336 |
| | 84.7 | 59.20000000000001 |
| 66 | 86.06 | 61.03333333333333 |
| | 88.34 | 62.233333333333334 |
| 72 | 89.4 | 64.63333333333333 |Time (h)
