# Supplementary material for: New Target Genes of MITF-Induced microRNA-211 Contribute to Melanoma Cell Invasion
Source: PLoS One. 2013 Sep 5;8(9):e73473. doi: 10.1371/journal.pone.0073473 (PMC3764006; doi:10.1371/journal.pone.0073473)
Supplement: Table S1 — Oligonucleotides and Primers for 3′ UTR cloning (Luciferase assays) and qPCR Primers. (DOCX) [file pone.0073473.s007.docx]

**Table S1. Oligonucleotides and Primers for 3’ UTR cloning (Luciferase assays) and qPCR Primers**

| **Oligos** | **5´-Sequence-3´** | |
| --- | --- | --- |
| 211-FC-F | 5’XhoI-TCGACAGGCGAAGGATGACAAAGGGAAT-3’XbaI | |
| 211-FC-R | 5’XbaI-CTAGATTCCCTTTGTCATCCTTCGCCTG-3’XhoI | |
| AP1S2-211BS1-F | 5’XhoI-TCGACCACAATTTTTTTTTAAAAGGGAACTTTT-3’XbaI | |
| AP1S2-211BS1-R | 5’XbaI-CTAGAAAAGTTCCCTTTTAAAAAAAAATTGTGG-3’XhoI | |
| AP1S2-211BS2-F | 5’XhoI-TCGACTGTAAAAAAAAAAAAAAAGGGAATTGAT-3’XbaI | |
| AP1S2-211BS2-R | 5’XbaI-CTAGATCAATTCCCTTTTTTTTTTTTTTTACAG-3’XhoI | |
| IGFBP5-211BS1-F | 5’XhoI-TCGACCATTTCATCTCATTTAAGGGAAAAATAT-3’XbaI | |
| IGFBP5-211BS1-R | 5’XbaI-CTAGATATTTTTCCCTTAAATGAGATGAAATGG-3’XhoI | |
| IGFBP5-211BS2-F | 5’XhoI-TCGACGTTGGATGAACAGAGAAGGGAAGAGAGT-3’XbaI | |
| IGFBP5-211BS2-R | 5’XbaI-CTAGACTCTCTTCCCTTCTCTGTTCATCCAACG-3’XhoI | |
| IGFBP5-211BS3-F | 5’XhoI-TCGACGAACGAGAGGAAGAGAAGGGAAGGAAGT-3’XbaI | |
| IGFBP5-211BS3-R | 5’XbaI-CTAGACTTCCTTCCCTTCTCTTCCTCTCGTTCG-3’XhoI | |
| IGFBP5-211BS4-F | 5’XhoI-TCGACTCCTTTTGGATGGGGAAAGGGAAGGTGT-3’XbaI | |
| IGFBP5-211BS4-R | 5’XbaI-CTAGACACCTTCCCTTTCCCCATCCAAAAGGAG-3’XhoI | |
| KCNMA1-211BS1-F | 5’XhoI-TCGACCAATTAATTGTAATTAAGGGAAATGAAT-3’XbaI | |
| KCNMA1-211BS1-R | 5’XbaI-CTAGATTCATTTCCCTTAATTACAATTAATTGG-3’XhoI | |
| KCNMA1-211BS2-F | 5’XhoI-TCGACTTTGTTCATATTTTTAAAGGGAAGACCT-3’XbaI | |
| KCNMA1-211BS2-R | 5’XbaI-CTAGAGGTCTTCCCTTTAAAAATATGAACAAAG-3’XhoI | |
| LIFR-211BS1-F | 5’XhoI-TCGACGGGATGTTTTCTTCCAAAGGGAATTTAT-3’XbaI | |
| LIFR-211BS1-R | 5’XbaI-CTAGATAAATTCCCTTTGGAAGAAAACATCCCG-3’XhoI | |
| M6PR-211BS1-F | 5’XhoI-TCGACCACAGGGGTTCCAGAAAAGGGAAGTCAT-3’XbaI | |
| M6PR-211BS1-R | 5’XbaI-CTAGATGACTTCCCTTTTCTGGAACCCCTGTGG-3’XhoI | |
| M6PR-211BS2-F | 5’XhoI-TCGACTTTTCAATCTTCTAGAAAGGGAACGGAT-3’XbaI | |
| M6PR-211BS2-R | 5’XbaI-CTAGATCCGTTCCCTTTCTAGAAGATTGAAAAG-3’XhoI | |
| PDE3A-211BS1-F | 5’XhoI-TCGACGTGAAAGAGAGACAGAAGGGAATGGTTT-3’XbaI | |
| PDE3A-211BS1-R | 5’XbaI-CTAGAAACCATTCCCTTCTGTCTCTCTTTCACG-3’XhoI | |
| RAB22A-211BS1-F | 5’XhoI-TCGACGAATCTCTAGTGTACAAAGGGACTACAT-3’XbaI | |
| RAB22A-211BS1-R | 5’XbaI-CTAGATGTAGTCCCTTTGTACACTAGAGATTCG-3’XhoI | |
| RAB22A-211BS2-F | 5’XhoI-TCGACTTATGTAACATTTGTAAAGGGAAAATTT-3’XbaI | |
| RAB22A-211BS2-R | 5’XbaI-CTAGAAATTTTCCCTTTACAAATGTTACATAAG-3’XhoI | |
| RAB22A-211BS3-F | 5’XhoI-TCGACACTTTTGTGGTTCTTAAGGGAAAAGAAT-3’XbaI | |
| RAB22A-211BS3-R | 5’XbaI-CTAGATTCTTTTCCCTTAAGAACCACAAAAGTG-3’XhoI | |
| RAB22A-211BS4-F | 5’XhoI-TCGACTATTCTTAAATATCTAAAGGGAAAGGAT-3’XbaI | |
| RAB22A-211BS4-R | 5’XbaI-CTAGATCCTTTCCCTTTAGATATTTAAGAATAG-3’XhoI | |
| RAB22A-211BS5-F | 5’XhoI-TCGACATTTACATTTGCTTTAAAGGGACAGTTT-3’XbaI | |
| RAB22A-211BS5-R | 5’XbaI-CTAGAAACTGTCCCTTTAAAGCAAATGTAAATG-3’XhoI | |
| SERINC3-211BS1-F | 5’XhoI-TCGACCTAAGGATATAGCACAAAGGGAACATTT-3’XbaI | |
| SERINC3-211BS1-R | 5’XbaI-CTAGAAATGTTCCCTTTGTGCTATATCCTTAGG-3’XhoI | |
| SOX4-211BS1-F | 5’XhoI-TCGACAAGAGTTTAAAGAGAAAAGGGAAAAAAT-3’XbaI | |
| SOX4-211BS1-R | 5’XbaI-CTAGATTTTTTCCCTTTTCTCTTTAAACTCTTG-3’XhoI | |
| SOX4-211BS2-F | 5’XhoI-TCGACGTGCAATTACAGCAAAAAGGGATTCTGT-3’XbaI | |
| SOX4-211BS2-R | 5’XbaI-CTAGACAGAATCCCTTTTTGCTGTAATTGCACG-3’XhoI | |
| SOX11-211BS1-F | 5’XhoI-TCGACTTTTTTTTAACAAAAAAAGGGACCATTT-3’XbaI | |
| SOX11-211BS1-R | 5’XbaI-CTAGAAATGGTCCCTTTTTTTGTTAAAAAAAAG-3’XhoI | |
| SOX11-211BS2-F | 5’XhoI-TCGACATTTTTGTACAGTGAAAAGGGAACATTT-3’XbaI | |
| SOX11-211BS2-R | 5’XbaI-CTAGAAATGTTCCCTTTTCACTGTACAAAAATG-3’XhoI | |
| SOX11-211BS3-F | 5’XhoI-TCGACTATAAAACGGCTTACAAAGGGAGACACT-3’XbaI | |
| SOX11-211BS3-R | 5’XbaI-CTAGAGTGTCTCCCTTTGTAAGCCGTTTTATAG-3’XhoI | |
| SSRP1-211BS1-F | 5’XhoI-TCGACTGAAAATGATTTAATAAAGGGAACTGAT-3’XbaI | |
| SSRP1-211BS1-R | 5’XbaI-CTAGATCAGTTCCCTTTATTAAATCATTTTCAG-3’XhoI | |
| **Primers** | **5´-Sequence-3´** |  |
| RAB22A-Nhe-F | AAAGCTAGCAATGGACCAGTTCTG |  |
| RAB22A-Xho-R | AAACTCGAGATGATACAACTCCCT |  |
| RAB22A-Xho-F | ATTCTCGAGTTGTTTGAAGTCCCT |  |
| RAB22A-Sbf-R | AAACCTGCAGGAGTTAACAGCTTGAC |  |
| AP1S2-Nhe-F | AATGCTAGCTGTAGATGGTCACTC |  |
| AP1S2-Sbf-R | ATTCCTGCAGGTTCCAATACAAGTCT |  |
| SERINC3-Nhe-F | AAAGCTAGCCTTCAGCTGAACCTC |  |
| SERINC3-Sbf-R | AAACCTGCAGGCTCAAATATCCAACA |  |
| **qPCR Primers** | **5´-Sequence-3´** |  |
| miR-211 | miScript Primer assay from QIAGEN |  |
| miR-204 | miScript Primer assay from QIAGEN |  |
| RNU1A | miScript Primer assay from QIAGEN |  |
| RNU5A | miScript Primer assay from QIAGEN |  |
| SCARNA17 | miScript Primer assay from QIAGEN |  |
| RAB22A-F | GCTCAAAGTGTGTCTGCTCG |  |
| RAB22A-R | AAAGATGCCCCTATTGTTGG |  |
| AP1S2-F | GCAGGAGGAAGCTGAAACC |  |
| AP1S2-R | GCTCAAAGTGTGTCTGCTCG |  |
| SERINC3-F | GCCATGTCCAATGAACCTG |  |
| SERINC3-R | CTCTTTGATGGTGGAGTAGGG |  |
| M6PR-F | CCACGACACGATGTTCCC |  |
| M6PR-R | CTGTCTGCCAGGATTCTCTCA |  |
| IGFBP5-F | TGACCGCAAAGGATTCTACA |  |
| IGFBP5-R | GGCACTGAAAGTCCCCGT |  |
| PDE3A-F | ATGAACAGGGTGATGAAGAGG |  |
| PDE3A-R | CCACAATGTGAGAGATGAAGGA |  |
| SSRP1-F | CGTCCACAAGCCACCTGT |  |
| SSRP1-R | ACTCCTCCCTCTCAATGCTG |  |
| SOX11-F | AGAAGATCCCGTTCATCCG |  |
| SOX11-R | CATTTTGGGCTTTTTCCG |  |
| LIFR-F | CAGTGGCTGTCATTGTTGGA |  |
| LIFR-R | TCTGGATTTGGAATATCAGGG |  |
| NR3C1-F | GCAATACCAGGTTTCAGGAAC |  |
| NR3C1-R | CCAGAGCAAATGCCATAAGA |  |
| ANGPT1-F | CCAAAGAGGCTGGAAGGAA |  |
| ANGPT1-R | GTCATACTGTGAATAGGCTCGG |  |
| SERP1-F | GAAGCACAGCAAGAACATCAC |  |
| SERP1-R | CCAATAACCAGGGTCCTACAG |  |
| TBP-F | ACCCAGCAGCATCACTGTT |  |
| TBP-R | CGCTGGAACTCGTCTCACTA |  |
| HPRT-F | TGGACAGGACTGAACGTCTT |  |
| HPRT-R | GAGCACACAGAGGGCTACAA |  |
| Cyclophilin A-F | CAGACAAGGTCCCAAAGACA |  |
| Cyclophilin A-R | CCATTATGGCGTGTGAAGTC |  |

Oligos representing miR-211 binding sites in the respective 3´UTRs of tentative target genes were annealed and cloned into pmirGLO vector as described in Materials and Methods. Primers were used to amplify larger regions covering binding sites, with the restriction enzymes indicated in their names. Primers used for qPCR amplifications are also listed.
